# Transcriptional and Epigenetic Regulation of *KIF14* Overexpression in Ovarian Cancer

**DOI:** 10.1371/journal.pone.0091540

**Published:** 2014-03-13

**Authors:** Brigitte L. Thériault, Halesha D. Basavarajappa, Harvey Lim, Sanja Pajovic, Brenda L. Gallie, Timothy W. Corson

**Affiliations:** 1 Campbell Family Cancer Research Institute, Princess Margaret Cancer Centre, Toronto, Ontario, Canada; 2 Eugene and Marilyn Glick Eye Institute, Department of Ophthalmology, and Department of Biochemistry and Molecular Biology, Indiana University School of Medicine, Indianapolis, Indiana, United States of America; 3 Department of Medical Biophysics, University of Toronto, Toronto, Ontario, Canada; 4 Division of Visual Science, Toronto Western Hospital Research Institute, Toronto, Ontario, Canada; 5 Departments of Molecular Genetics and Ophthalmology, University of Toronto, Toronto, Ontario, Canada; 6 Indiana University Melvin and Bren Simon Cancer Center, Indianapolis, Indiana, United States of America; Institut de Génomique Fonctionnelle de Lyon, France

## Abstract

*KIF14* (kinesin family member 14) is a mitotic kinesin and an important oncogene in several cancers. Tumor *KIF14* expression levels are independently predictive of poor outcome, and in cancer cells KIF14 can modulate metastatic behavior by maintaining appropriate levels of cell adhesion and migration proteins at the cell membrane. Thus KIF14 is an exciting potential therapeutic target. Understanding KIF14's regulation in cancer cells is crucial to the development of effective and selective therapies to block its tumorigenic function(s). We previously determined that close to 30% of serous ovarian cancers (OvCa tumors) exhibit low-level genomic gain, indicating one mechanism of *KIF14* overexpression in tumors. We now report on transcriptional and epigenetic regulation of *KIF14*. Through promoter deletion analyses, we identified one *cis*-regulatory region containing binding sites for Sp1, HSF1 and YY1. siRNA-mediated knockdown of these transcription factors demonstrated endogenous regulation of *KIF14* overexpression by *Sp1* and *YY1*, but not *HSF1*. ChIP experiments confirmed an enrichment of both Sp1 and YY1 binding to the endogenous KIF14 promoter in OvCa cell lines with high *KIF14* expression. A strong correlation was seen in primary serous OvCa tumors between *Sp1*, *YY1* and *KIF14* expression, further evidence that these transcription factors are important players in *KIF14* overexpression. Hypomethylation patterns were observed in primary serous OvCa tumors, suggesting a minor role for promoter methylation in the control of *KIF14* gene expression. miRNA expression analysis determined that miR-93, miR-144 and miR-382 had significantly lower levels of expression in primary serous OvCa tumors than normal tissues; treatment of an OvCa cell line with miRNA mimics and inhibitors specifically modulated *KIF14* mRNA levels, pointing to potential novel mechanisms of *KIF14* overexpression in primary tumors. Our findings reveal multiple mechanisms of KIF14 upregulation in cancer cells, offering new targets for therapeutic interventions to reduce KIF14 in tumors, aiming at improved prognosis.

## Introduction

*KIF14* was first identified as an oncogene and a contributor to malignant transformation in the childhood cancer retinoblastoma [1]. Located on chromosome 1q32.1, genomic gain of *KIF14* occurs in up to 50% of retinoblastomas [1]–[3]. Our group and others have previously shown that KIF14 protein and mRNA are overexpressed in multiple cancers including ovarian cancers (OvCa tumors) [4]–[10]. We also reported that overall outcome of serous OvCa patients can be predicted based on *KIF14* mRNA expression levels in their primary tumors. Further analysis of these samples showed that expression of *KIF14* mRNA and protein exceed the levels expected based on the copy number gain alone, suggesting an up-regulation in the transcriptional control in cancer cells versus their respective normal counterparts.

There are currently 45 human kinesins classified into 14 distinct families. In humans, *KIF14* is a mitotic kinesin belonging to the N-3 family. Although its cellular function has not yet been fully elucidated, *KIF14* plays a vital role in the completion of cytokinesis, and may also function in the primary cilium [11]. It interacts with protein-regulator of cytokinesis 1 (PRC1) and citron kinase through specific domains to support proper cell division [12], [13]. Silencing *KIF14* using siRNA induces cytokinesis failure resulting in multinucleated cells, aneuploidy, and apoptosis [12]. A temporary accumulation of KIF14 protein is observed in mitotic cells, consistent with its function [12]. We recently demonstrated in breast cancer cells lines, direct interaction of KIF14 with Radil, a crucial mediator of Rap1a-mediated integrin inside-out signaling, thereby controlling Radil-Rap1a activity at the cell membrane and promoting cell adhesion and migration [14].

Whereas the structure and function of other kinesins are fairly well understood, much less is known about their regulation at the DNA level. One study described roles of the transcription factors Sp1 and E2F1 in respectively activating and repressing the transcription of the human mitotic centromere-associated kinesin (MCAK) promoter [15]. No studies have yet been reported on *KIF14*. Understanding the mechanism of its gene regulation is crucial as *KIF14* emerges as an important target in cancer therapy.

We now report identification of a putative *cis*-regulatory region of the *KIF14* promoter harbouring binding sites for the transcription factors *Sp1*, *YY1* and *HSF1*. Through siRNA knockdown and ChIP assays, we show that Sp1 and YY1, but not HSF1 directly bind to the *KIF14* promoter and control endogenous *KIF14* levels in OvCa cell lines. Furthermore, both *Sp1* and *YY1* expression levels correlate with *KIF14* mRNA expression in primary serous OvCa tumors, demonstrating their potential role in maintaining high KIF14 levels in OvCa tumor cells. Methylation analysis revealed that the human *KIF14* promoter is largely hypomethylated in OvCa primary tumors, normal ovary tissues, and cell lines, indicating that methylation is unlikely to regulate *KIF14* overexpression within OvCa tumors. However, a differential methylation pattern may exist in OvCa tumors expressing very high KIF14.

miRNA expression analysis of primary OvCa tumors and cell lines revealed three putative regulators (miR-93, miR-144 and miR-382) of *KIF14* expression that have documented roles in tumorigenesis in cancer cells. We determined that these candidate miRNAs could directly modulate *KIF14* mRNA expression, indicating their importance in maintaining elevated KIF14 tumor levels. Our results unveil a complexity of regulatory mechanisms driving *KIF14* overexpression in OvCa tumors, and underscore the importance of understanding *KIF14* regulation to ultimately target this gene for therapeutic benefit.

## Materials and Methods

### Construction of Luciferase Reporter Plasmids

Sixteen different lengths of the *KIF14* promoter (548 bp, 966 bp, 1514 bp, 1666 bp, 1787 bp, 1898 bp, 2032 bp, 2150 bp, 2245 bp, 2327 bp, 2366 bp, 2401 bp, 2466 bp, 2536 bp, 2898 bp, 4555 bp) all sharing common sequences at their proximal end were amplified by polymerase chain reaction (PCR) from genomic DNA of healthy retina using KOD Hot Start DNA Polymerase (Novagen) (Table S1). Promoter fragments were subcloned into StrataClone™ Blunt PCR Cloning Vector pSC-B (Stratagene) and verified for correct insertion and orientation by restriction digest. PCR product was excised out using Kpn1/Sma1 restriction sites and inserted into pGL3-Basic vector (Promega) at complementary sites upstream of the luciferase gene generating the reporter constructs pGL3-548, pGL3-966, pGL3-1514, pGL3-1666, pGL3-1787, pGL3-1898, pGL3-2032, pGL3-2150, pGL3-2245, pGL3-2327, pGL3-2366, pGL3-2401, pGL3-2466, pGL3-2536, pGL3-2898, and pGL3-4555. Sequences of all 16 constructs aligned with the reference sequences from the NCBI website. The pRSV-Luciferase reporter plasmid was kindly provided by Dr. A. Schimmer, Ontario Cancer Institute.

### Cell Culture

SKOV3 (a kind gift from Dr. Mark Nachtigal, University of Manitoba) [16] and HeLa cells (ATCC) were cultured in Dulbecco's Modified Eagle's Medium (DMEM) containing 10% fetal bovine serum (FBS) and penicillin/streptomycin/L-glutamine (P/S/G) (Invitrogen). OvCa 429 cells (Dr. Mark Nachtigal) [16] were cultured in alpha-MEM containing 10% FBS and P/S/G. WERI-Rb1 retinoblastoma cells (positive control for Sp1 binding) were cultured in Iscove's modified Dulbecco's medium (IMDM) containing 10% FBS (PAA laboratories), P/S/G, 10 μg/mL insulin, and 55 μM β-mercaptoethanol. Cell cultures were maintained at 5% CO_2_ with humidity in a 37°C incubator. Cell line identity was confirmed by short tandem repeat profiling.

### Transfection of Reporter Constructs and siRNA

Trypsinized cells were seeded at a density of 5×10^4^ cells/mL. 24 hours after seeding, equal amounts (0.5 μg) of both reporter and cytomegalovirus-β-galactosidase (pCMV-β-gal, Promega) constructs were cotransfected into triplicate 12-well plates using Gene Juice (Novagen). To evaluate the endogenous effect of transcription factor knockdown on *KIF14* levels, a set of 3 siRNAs (Sigma Aldrich) for *HSF1*, *Sp1*, and *YY1* were transfected at an amount of 0.5 nmol into 60 mm dishes. Target sequences for each siRNA are listed in Table S2. Transfections were carried out in triplicate according to the manufacturers' instructions and repeated at least three times.

### Luciferase and β-galactosidase Assay

Cells that were cotransfected with reporter and pCMV-β-gal constructs were harvested 24 hours after transfection. Cell lysates were prepared and assayed for luciferase activity using the Luciferase Assay System (Promega) in accordance to its protocol. Luciferase activity was normalized to β-galactosidase activity (measured by hydrolysis of the colorimetric substrate ONPG [*O*-nitrophenyl β-d-galactopyranoside], Sigma) and expressed as a relative percentage of pRSV-Luc control.

### Clinical Samples

Twenty-six fresh frozen OvCa tumor samples, ten fresh frozen normal non-neoplastic ovaries from patients undergoing oophorectomy for non-oncological conditions and four normal tubal epithelium tissues were obtained from the University Health Network (UHN) Biobank. The UHN Research Ethics Board approved the study of tissue samples and associated clinical data (#08-0884-TE), and all tissues were banked with written informed consent. Clinical data associated with the UHN Biobank samples were reviewed by a gynecological oncologist and gynecologic pathologist to ensure data integrity and quality, and all UHN Biobank tissues (adjacent H&E stained slides) were reviewed by a gynecologic pathologist to ensure samples contained >80% tumor cells.

### Real-time PCR

Seventy-two hours following transfection with siRNA, cell lines were harvested and total RNA isolated by TRIzol reagent (Invitrogen) according to manufacturer's instructions. OvCa tumor and normal ovary and tubal epithelial RNAs were also isolated by TRIzol reagent as previously described [8]. First-strand cDNA was prepared from 1 μg of total cellular RNA with 100 μM random hexamer primers, 20 U RiboLock RNase Inhibitor (Fermentas), and 200 U Superscript II reverse transcriptase (Invitrogen) in a final volume of 20 μL. 1 μL of synthesized cDNA was added to 1X TaqMan PCR master mix (ABI), and 1X TaqMan Gene Expression Assay primer-probe mix for *KIF14* (Hs00978216_m1), *Sp1* (Hs00916521_m1), *YY1* (Hs00231533_m1), and *HSF1* (Hs01027608_g1). Mean expression of three housekeeping genes was used as an endogenous control: *TBP* (Tata-box binding protein, Hs_99999910_m1), *HPRT* (Hypoxanthine phosphoribosyl transferase, Hs99999909_m1) and *GAPDH* (glyceraldehyde-3-phosphate dehydrogenase, Hs99999905_m1). Triplicate reactions were conducted for each gene and each sample, and PCR performed using the SDS 7900HT system (ABI) as described [8]. SDS 2.1 software (ABI) was used to calculate the ΔΔCt relative expression values, normalized to endogenous control genes and relative to either vector control (for cell line siRNA transfections) or expression of normal ovary and tubal epithelial tissue samples (for tissue measurements).

### Western Blot Analysis

Seventy-two hours after transfecting siRNA, cells were harvested and lysed with buffer containing 20 mM Tris HCl pH 7.6, 150 mM NaCl, 1 mM ethylenediaminetetraacetic acid (EDTA), 1% Triton X-100, and the protease inhibitors aprotinin, leupeptin, and phenylmethanesulphonylfluoride (PMSF). Total cellular protein was quantified by Bradford assay (BioRad). 30 μg was loaded onto a 4–12% gradient SDS-PAGE precast gel (Lonza) and transferred onto a polyvinylidene difluoride (PVDF) membrane and blocked with 5% BLOTTO (BioRad) in Tris-Buffered Saline-0.05% Tween-20 (TBST). Primary antibodies against KIF14 (Bethyl), Sp1, YY1 and HSF1 (Abcam) and β-tubulin (Sigma) were employed to detect endogenous protein expression. Horseradish peroxidase-labeled secondary antibodies (Chemicon) were detected using a chemiluminescence reagent (Denville) and incubated with photographic film (Denville). Signal intensity measurements were calculated using Photoshop CS3.

### Chromatin Immunoprecipitation (ChIP) Assay

ChIP assays were conducted with the Imprint® Chromatin Immunoprecipitation Kit (Sigma) according to manufacturer instructions. Briefly, 1×10^5^ cells were cultured, and the protein-DNA complexes cross-linked with 1% formaldehyde (final concentration), followed by nuclear fractionation and DNA shearing via sonication. Immunoprecipitations were carried out for 90 minutes at RT with antibodies against Sp1, YY1, HSF1 (Abcam), RNA polymerase II (positive control) and IgG (negative control). The cross-links were reversed, DNA was extracted and was used for both endpoint and real time PCR. Endpoint PCR was performed in 25 μL reactions with KOD Hot Start DNA Polymerase (Novagen) and the appropriately designed primer pair (target promoter sequence: Forward: tta caa tgt gaa gtc ttc gat gta; Reverse: ctc tac tcc, cac ccc gc; RNA Pol II target sequence: hGAPDH-Forward: caa ttc ccc atc tca gtc gt; hGAPDH-Reverse: tag tag ccg ggc cct act tt, 246 bp). The PCR conditions were as follows: 95°C for 2 min followed by 40 cycles of denaturation at 95°C for 30 sec, annealing at 58°C for 30 sec, and extension at 72°C for 30 sec, and then a final extension period of 72°C for 10 min. Products were visualized using agarose gel electrophoresis. Signal intensity measurements were calculated using Photoshop CS3. Real time PCR was performed in triplicate using 1 μL of ChIP DNA per reaction, along with 1X TaqMan Master Mix (ABI), 500 nM each primer and 250 nM probe. Primers were Forward: tga cac cca ctt caa cga gg and Reverse: tct ctg aat gct gga ctc gc, and the probe was cac gct tta gca gaa ccc gag gag, labeled with FAM and ZEN quencher (Integrated DNA Technologies, Coralville, IA, USA). Reactions were run using standard parameters on a ViiA7 instrument (Life Technologies). Ct values were normalized to IgG samples and relative quantity (RQ) calculated according to RQ = 2^−ΔCt^.

### miRNA cDNA synthesis and quantitative real-time PCR

Total RNA extraction was performed as described above. A commercially available miRNA cDNA synthesis kit (TaqMan® MicroRNA Reverse Transcription Kit, Applied Biosystems) was used to reverse transcribe the candidate miRNAs with specific RT primers (TaqMan® miRNA Assays, Applied Biosystems). Sequence-specific assays (TaqMan® real-time PCR assays, Applied Biosystems, Table S3) were used to detect mature candidate miRNAs from primary tumor tissue extracts. *RNU44*, a small non-coding RNA with wide and constant tissue distribution (Applied Biosystems), was used as the endogenous control. Triplicate reactions were conducted for each gene and each sample, and PCR performed using the SDS 7900HT system (ABI) as described [8]. SDS 2.1 software (ABI) was used to calculate the ΔΔCt relative expression values, normalized to endogenous control (*RNU44*) and relative to expression of normal ovary and tubal epithelial tissue samples (for tumor tissue measurements) or IOSE cells (for OvCa cell lines).

### miRNA mimics and inhibitors

SKOV3 cells in 12-well plates were transfected with 50 nM of miR93, miR144 and miR382 mimics or inhibitors or corresponding non-coding control mimic or inhibitor (Applied Biosystems) using 3 μL of Lipofectamine 2000 (Invitrogen) reagent as per the manufacturer's instructions. After 72 hours of transfection, total RNA was isolated and cDNA synthesized as described above. Expression levels of KIF14 and miRNAs of interest were analyzed as above, with qPCR performed using an Applied Biosystems ViiA™ 7 real time PCR system. The relative expression values (RQ) of KIF14 or miRNAs normalized to endogenous control genes and relative to control treatment were calculated using ViiA™ 7 version 1.2 software.

### Methylation analysis

Genomic DNA was isolated from primary OvCa tumor tissues and normal ovary controls as previously described [8]. Genomic DNA (200 ng) was modified and purified using a commercially available bisulphite modification kit (Imprint® DNA Modification Kit, Sigma) according to manufacturer's instructions. A CpG island was identified (http://cpgislands.usc.edu/) within the 4500 bp *KIF14* promoter region (−2371 to −1129) (Figure S1A), and methylation-specific primers (∼120 bp) against the largest CpG island within the *KIF14* promoter using the online software Methprimer were designed (http://www.urogene.org/methprimer/) (Figure S1B). Primer sequences were as follows: Methylated-specific: Forward: att taa agg ggg tta agt ttt acgt; Reverse: gat aat taa act ccg ata acc gtc; Unmethylated-specific: Forward: att taa agg ggg gtt agt ttt atgt; Reverse: caa taa tta aac tcc aat aac catc. One quarter of the purified bisulphite-treated DNA (5 μL of a 20 μL eluate) was used in the methylation PCR reaction. End-point PCR reactions were conducted in 25 μL final volume with Maxima Hot Start Taq Polymerase (Fermentas). The PCR conditions were as follows: 94°C for 3 min followed by 40 cycles of denaturation at 94°C for 30 sec, annealing at 60°C for 30 sec, and extension at 72°C for 30 sec, and then a final extension period of 72°C for 10 min. Products (∼120 bp) were visualized using agarose gel electrophoresis. Densitometry was performed on grayscale images using Image J. Verification of bisulphite conversion of the DNA was conducted with unmodified calponin-specific primers (Figure S2) as described [17].

### Statistical Analysis

Expression values (luciferase activity, mRNA, miRNA or protein expression) were assessed between normal tissue controls and OvCa tumor tissues, or controls and treated groups using paired or unpaired t-tests. KIF14 expression after miRNA inhibitor/mimic treatment was analyzed by one-way ANOVA. Unless otherwise specified, all statistical analyses were conducted using Graph Pad Prism 4.0. Correlations between *KIF14* gain vs. *KIF14* no gain categories and *Sp1*, *YY1* and *HSF1* mRNA expression were analyzed using Pearson's Correlation Coefficient (r).

## Results

### Identification of a *KIF14* cis regulatory region in ovarian cancer cell lines

**Figure 1:**
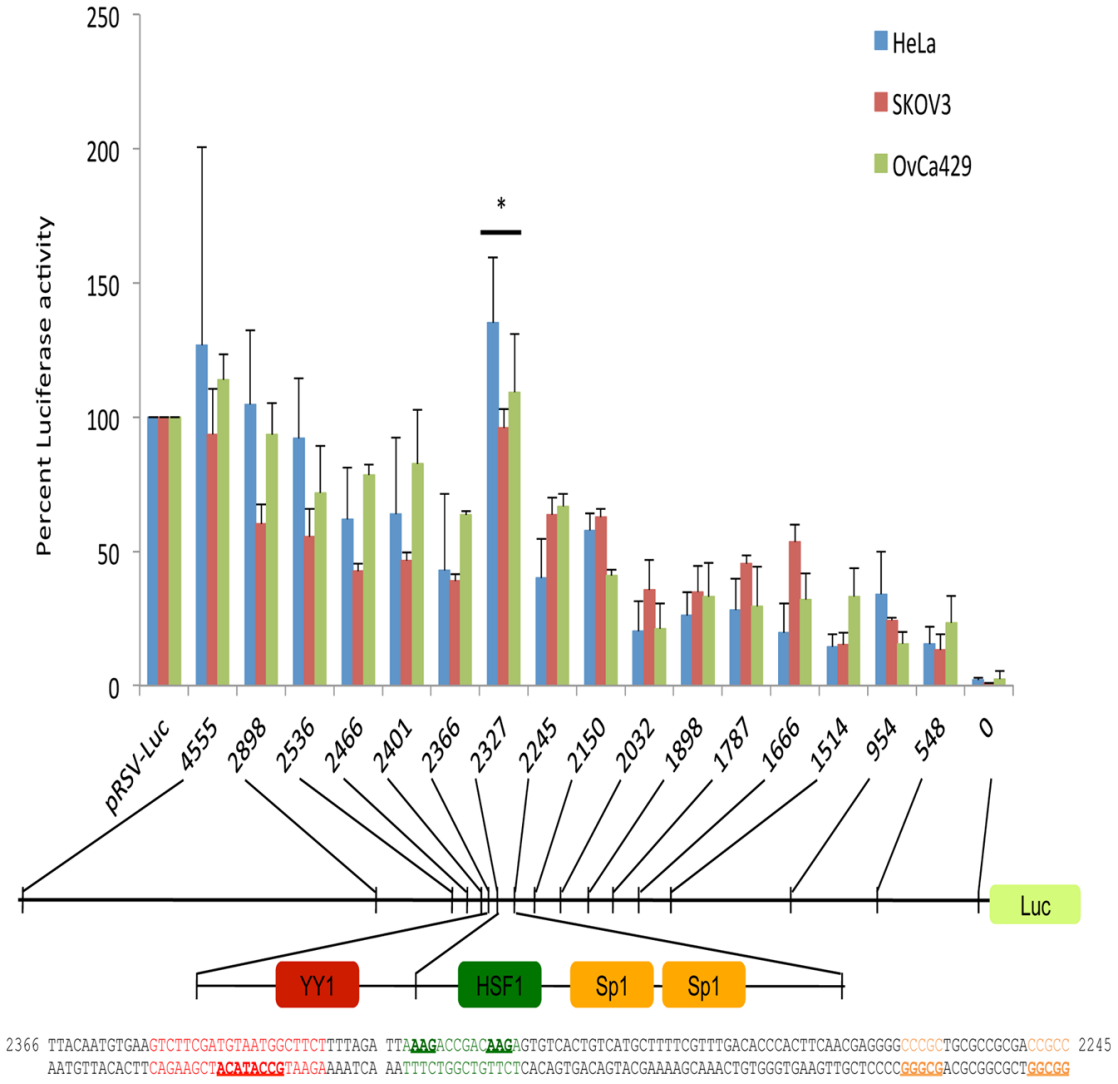
KIF14 promoter activity. Promoter deletion constructs fused to Luciferase were tested for activity in HeLa, SKOV3 and OvCa429 cells. pRSV-Luc, positive control, 0, empty vector control. Numbers are base pairs relative to the transcriptional start site (0). Bioinformatic analysis (Genomatix) of the most active region within the *KIF14* promoter (−2366 to −2245) identified putative binding sites for transcription factors YY1, HSF1 and Sp1. Specific transcription factor recognition sites are underlined, and space within sequence denotes location of deletion constructs. N = 3, * Significance at *P*<0.05, unpaired t-test. *P* = 0.01 (HeLa); *P* = 0.01 (SKOV3); *P* = 0.03 (OvCa429).

A 4500 bp region of genomic DNA immediately upstream of the human *KIF14* transcriptional start site was amplified from normal human DNA, and deletional reporter analyses were conducted by cloning sequentially smaller regions of the *KIF14* promoter into the pRSV-Luc promoter region. Luciferase activity was measured in OvCa429, SKOV3 and HeLa cells, and promoter activity was calculated in relation to pRSV-Luc expression (set at 100%). The entire promoter region construct (pGL3-4555) showed luciferase activity close to that of the positive control (pRSV-Luc). One highly active region was identified between -2366 and -2245 bp, demonstrating similar activity to the full promoter construct, but significantly more luciferase activity compared to all other deletion constructs for all three cell lines (P<0.05, Figure 1). Bioinformatic analysis of this active region through online analysis software (Genomatix) identified putative transcription factor binding sites for *Sp1*, *YY1* and *HSF1* (Figure 1), suggesting the presence of a potential *cis* regulatory region within the *KIF14* promoter.

### Sp1 and YY1 endogenously regulate KIF14 expression in OvCa cell lines

**Figure 2:**
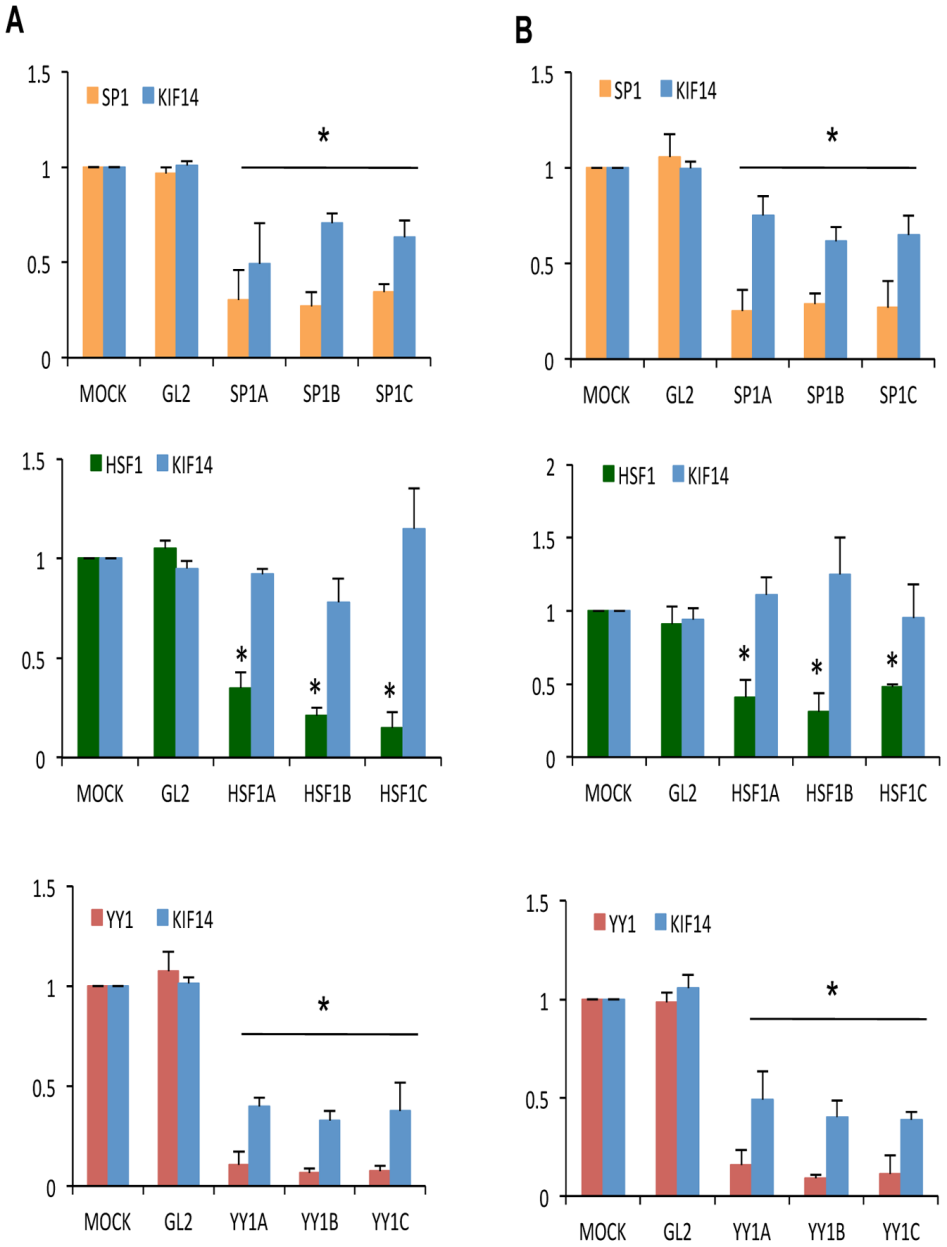
Knockdown of endogenous SP1 and YY1 mRNA inhibits KIF14 transcription. siRNA knockdown of endogenous *Sp1* (orange), *HSF1* (green), and *YY1* (red) transcription factors, and measurement of their mRNA expression along with corresponding *KIF14* mRNA levels (blue) via real-time PCR in **A** SKOV3 and **B** OvCa429 cells. Y-axes: normalized mRNA expression relative to MOCK. GL2, control siRNA; N = 3, * Significance at *P*<0.05, unpaired t-test. Three different siRNA molecules (A, B, C) were used to knock down each gene. P values for panel A (SKOV3 cells): *P* = 0.02 for Sp1 expression (orange) with Sp1 siRNAs A, B, and C; *P* = 0.03 (siRNA-A), *P* = 0.047 (siRNA–B), *P* = 0.04 (siRNA–C) for KIF14 expression (blue). *P* = 0.001 for HSF1 expression (green) with HSF1 siRNAs A, B, and C; *P* = 0.23 (siRNA-A), *P* = 0.12 (siRNA-B), *P* = 0.4 (siRNA-C) for KIF14 expression (blue). *P* = 0.01 for YY1 expression (red) with YY1 siRNAs A, B and C; *P* = 0.006 for KIF14 expression (blue) with YY1 siRNAs A, B, and C. P values for panel B (OvCa429 cells): *P* = 0.03 for Sp1 expression (orange) with Sp1 siRNAs A, B, and C; *P* = 0.05 (siRNA-A), *P* = 0.04 (siRNA-B), *P* = 0.045 (siRNA-C) for KIF14 expression (blue). *P* = 0.003 for HSF1 expression (green) with HSF1 siRNAs A, B, and C; *P* = 0.31 (siRNA-A), *P* = 0.45 (siRNA-B), *P* = 0.39 (siRNA-C) for KIF14 expression (blue). *P* = 0.02 (siRNA-A), *P* = 0.01 (siRNA-B), *P* = 0.01 for YY1 expression (red); *P* = 0.02 (siRNA-A), *P* = 0.001 (siRNA-B), *P* = 0.006 (siRNA-C) for KIF14 expression (blue).

**Figure 3:**
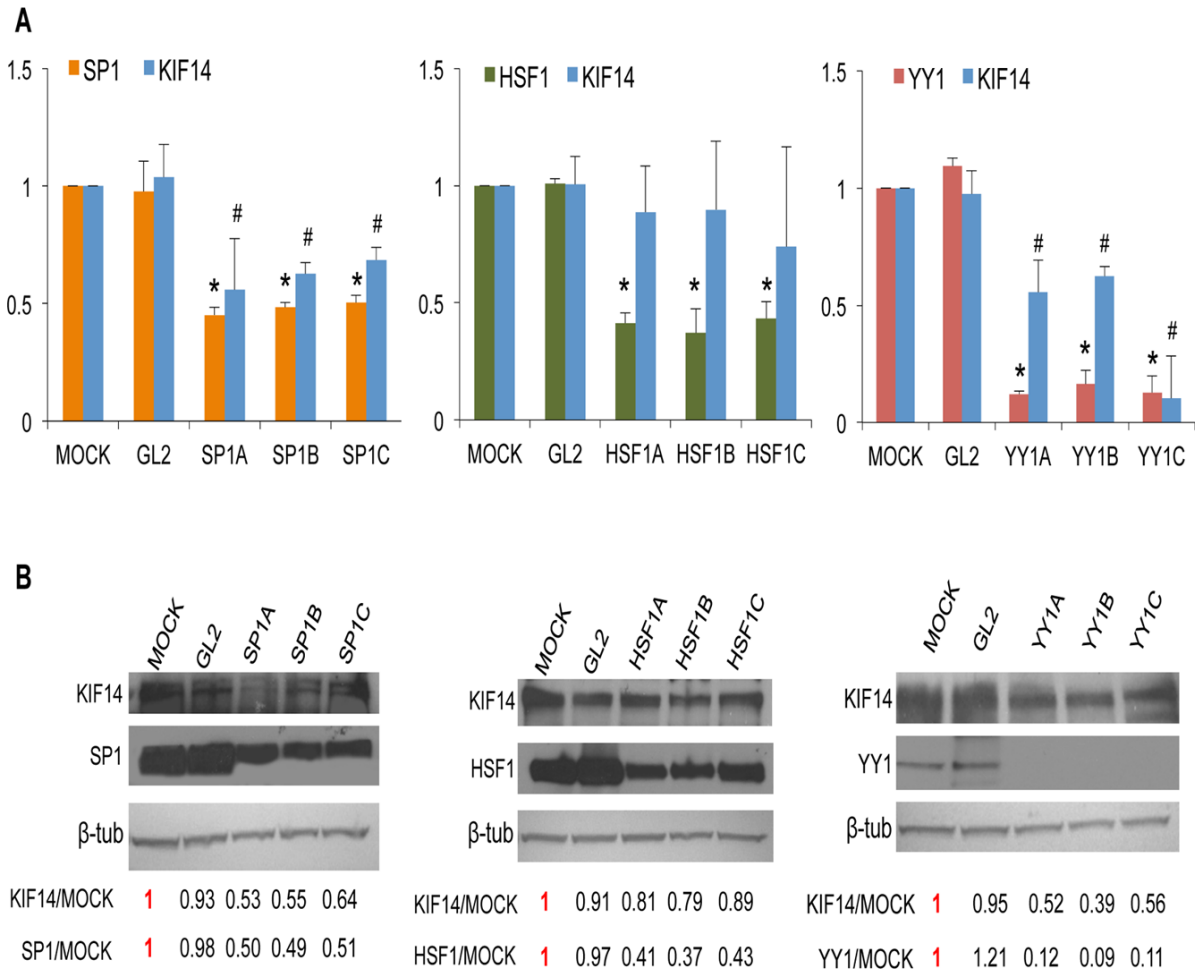
Knockdown of endogenous SP1 and YY1 protein inhibits KIF14 protein expression. A siRNA knockdown of endogenous Sp1 (orange), HSF1 (green), and YY1 (red) transcription factors, and measurement of their protein expression along with corresponding KIF14 protein levels (blue) via immunoblot in SKOV3 cells. x-axis: normalized protein expression relative to MOCK. B Representative immunoblot of KIF14 and transcription factor expression. Numbers represent normalized expression values for the experiment shown. Similar results were seen with OvCa429 cells. GL2, control siRNA; N = 3; *, *P*<0.05 for transcription factor expression; #, *P*<0.05 for KIF14 expression, unpaired t-test. *P* values for panel A (SKOV3 cells): *P* = 0.009 (siRNA-A), *P* = 0.003 (siRNA-B), *P* = 0.006 (siRNA-C) for Sp1 expression (orange); *P* = 0.005 (siRNA-A), *P* = 0.007 (siRNA-B), *P* = 0.004 (siRNA-C) for KIF14 expression (blue). *P* = 0.01 for HSF1 expression (green) with HSF1 siRNAs A, B, and C; *P* = 0.54 (siRNA-A), *P* = 0.65 (siRNA-B), *P* = 0.41 (siRNA-C) for KIF14 expression (blue). *P* = 0.001 for YY1 expression (red) with YY1 siRNAs A, B and C; *P* = 0.01 (siRNA-A), *P* = 0.02 (siRNA-B), *P* = 0.005 (siRNA-C) for KIF14 expression (blue).

We tested whether these transcription factors affected *KIF14* mRNA expression by siRNA-mediated knockdown of endogenous *Sp1*, *HSF1* and *YY1* in SKOV3 and OvCa429 cells. Transient knockdown resulted in a significant reduction of transcription factor (*Sp1*, *HSF1*, *YY1*) mRNA (Figure 2A, B). While knockdown of *HSF1* had no significant effect on *KIF14* gene expression, *Sp1* and *YY1* knockdown resulted in significantly decreased *KIF14* mRNA, suggesting that both Sp1 and YY1 act as enhancers of *KIF14* transcription. In response to *Sp1* and *YY1* knockdown, a decrease in transcription factor protein expression and associated KIF14 protein was confirmed via immunoblots (Figure 3) in SKOV3 cells. While HSF1 protein levels decreased in response to knockdown, no significant change in KIF14 protein expression was seen. Similar results were seen for OvCa429 cells (data not shown), indicating that *Sp1* and *YY1* may control the expression of *KIF14* in OvCa cell lines. The reduction in KIF14 mRNA and protein expression in response to Sp1 knockdown was less than with YY1 knockdown, and could likely be related to the efficiency of transcription factor knockdown (Figure 2A, B and Figure 3A, B). The reduction in *KIF14* mRNA and protein was more pronounced in response to *YY1* knockdown, suggesting that YY1 may be a major player in the regulation of *KIF14* overexpression in these cells.

**Figure 4:**
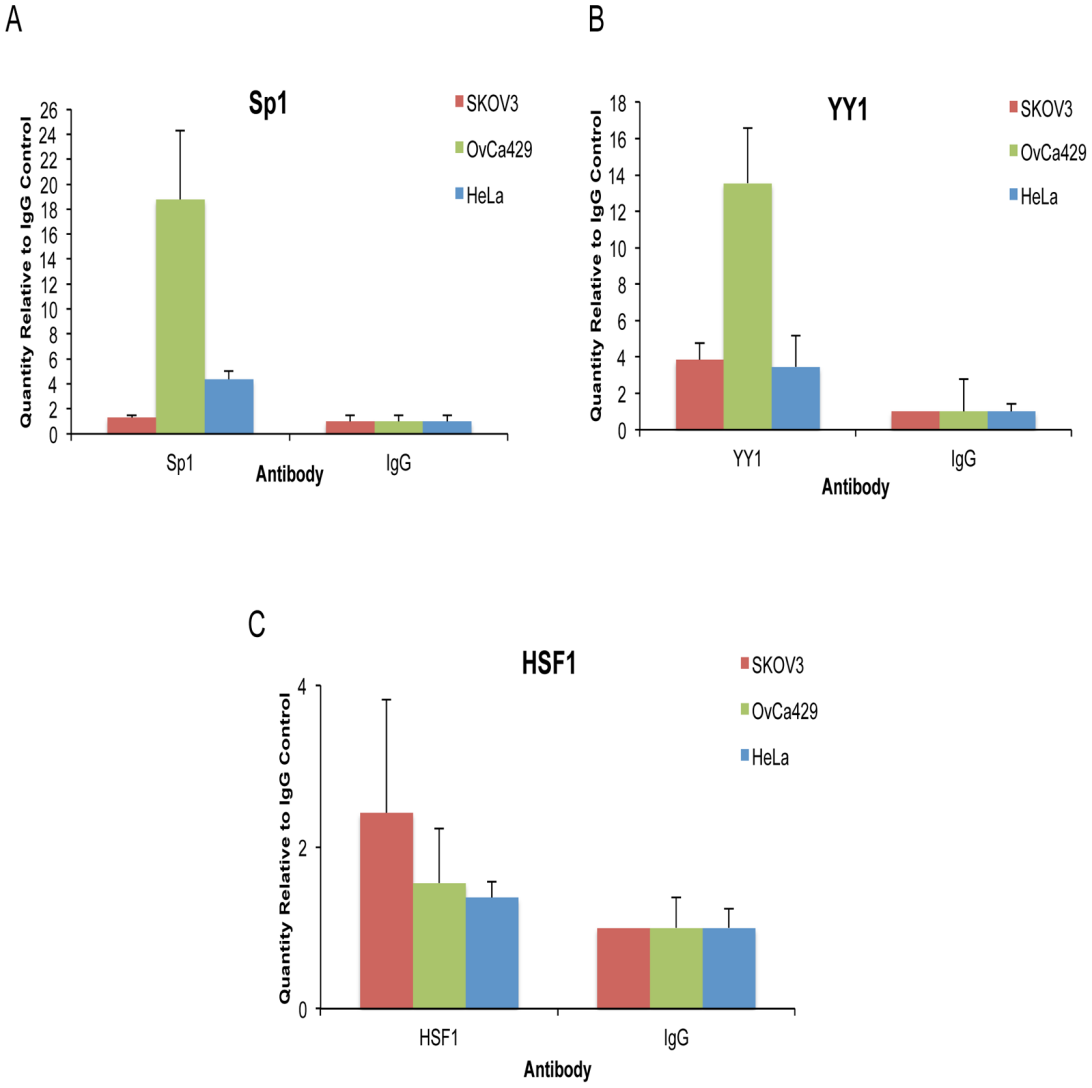
SP1 and YY1 bind endogenously to the human KIF14 promoter in cancer cell lines. ChIP assays of endogenous YY1, Sp1 and HSF1 followed by real time PCR with the *KIF14* promoter region (−2300 to −2133) in cell lines SKOV3, OvCa429, and HeLa compared to IgG (negative control). Values represent average quantity of promoter region product relative to IgG control. Error bars represent standard deviation of triplicate assays.

To verify whether Sp1 and YY1 could associate directly with the *KIF14* promoter, chromatin immunoprecipiation (ChIP) assays were conducted in SKOV3, OvCa429 and HeLa cells. We found that both Sp1 and YY1, but not HSF1 exhibited a much higher binding affinity to the *KIF14* promoter region than the IgG control (higher relative expression values; Figure 4). OvCa429 cells showed the greatest enrichment of binding for both Sp1 and YY1 (over 10-fold; Figure 4A, B), while SKOV3 and HeLa cells showed more modest enrichment (on average 4-fold; Figure 4A, B). Binding of HSF1 to the KIF14 promoter was much less pronounced (on average 2-fold for all cell lines; Figure 4C). Enrichment in Sp1 and YY1 binding was also shown via endpoint PCR (Figure S3). These results confirm our mRNA and protein expression results by showing that both Sp1 and YY1 can bind directly to the *KIF14* promoter. Combined, these data indicate that Sp1 and YY1 can endogenously regulate *KIF14* expression in OvCa cell lines.

### Sp1 and YY1 overexpression correlates with KIF14 overexpression

**Figure 5:**
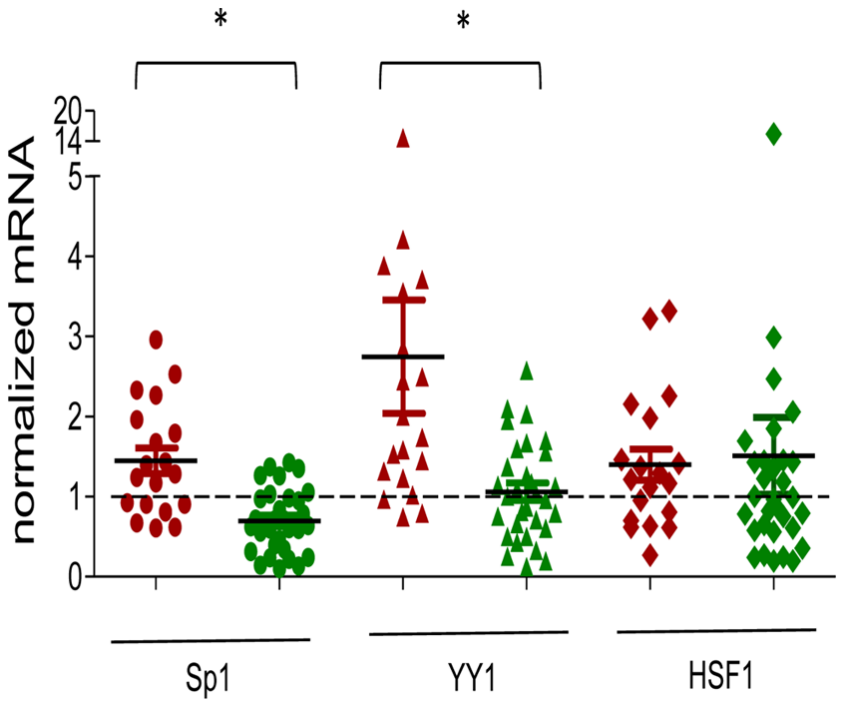
KIF14^HIGH^ OvCa tumors overexpress Sp1 and YY1. Quantitative mRNA expression analysis of primary OvCa tumors with *KIF14* ^HIGH^ (red) and *KIF14* ^LOW^ (green) mRNA expression (but no KIF14 genomic gain) for *Sp1* (circle), *YY1* (triangle), and *HSF1* (diamond) normalized to normal ovary expression (set as 1, black dashed line). Mean, black line. Individual tumors represented by symbols. * Significance at *P*<0.05, paired t-test. *P* = 0.03 (Sp1), *P* = 0.01 (YY1), *P* = 0.32 (HSF1).

We have previously documented genomic gain of *KIF14* in up to 30% of primary OvCa tumors that correlates with very high overexpression of *KIF14* mRNA (*KIF14* ^HIGH^) [8]. These data suggest that genomic gain is one mechanism through which *KIF14* is overexpressed in these tumors. To determine whether *KIF14* overexpression in OvCa tumors could also be linked to transcriptional regulation, we measured mRNA expression of *Sp1*, *YY1* and *HSF1* in a subset of OvCa tumor tissues with (15 samples) and without (50 samples) *KIF14* genomic gain. In OvCa tumors without genomic gain (50), we dichotomized the samples into two groups based on median expression, into either *KIF14* ^HIGH^ or *KIF14* ^LOW^ groups, as we have previously reported [8]. Many of the 19 *KIF14* ^HIGH^ OvCa tumors expressed significantly higher levels of *Sp1* and *YY1* mRNA (Figure 5) than those with *KIF14* ^LOW^ overexpression (31), indicating potential roles for Sp1 and YY1 to maintain high *KIF14* levels in tumors. *HSF1* mRNA levels were similar in all OvCa tumors, thus less likely to be important in regulating *KIF14* mRNA. The mean *YY1* expression level in primary OvCa tumors is much higher than the mean *Sp1* expression level; together with the *YY1* knockdown data in cell lines (Figures 2 and 3), these results suggest that YY1 is an important regulator of KIF14 overexpression in OvCa tumors. In support of this, a strong correlation was seen between *KIF14* and *Sp1* or *YY1* expression, whereas none existed for *HSF1* (Table 1 and Figure S4D–F). Tumors with KIF14 genomic gain demonstrated no transcription factor correlation (Table 1 and Figure S4A–C), confirming that genomic gain is the mechanism controlling *KIF14* overexpression in these tumors. Interestingly, a proportion (approximately 40%) of *KIF14* ^HIGH^ tumors showed *Sp1* and *YY1* expression close to normal tissue levels (Figure 5), further implicating other biological mechanisms to potentially control *KIF14* overexpression in OvCa tumors.

**Table 1:** Pearson correlation between *KIF14* gain/no gain tumors and *Sp1*, *HSF1* and *YY1* mRNA.

gain	Sp1	HSF1	YY1
r	−0.07	−0.13	−0.08
*P*	0.8	0.62	0.77
N	15	15	15

no gain	Sp1	HSF1	YY1
r	0.4	0.2	0.35
*P*	0.004	0.06	0.01
N	50	50	50

r =  correlation coefficient; *P* significance at <0.05; N =  number of samples.

### The *KIF14* promoter is differentially hypomethylated in primary serous OvCa tumors and cell lines

**Figure 6:**
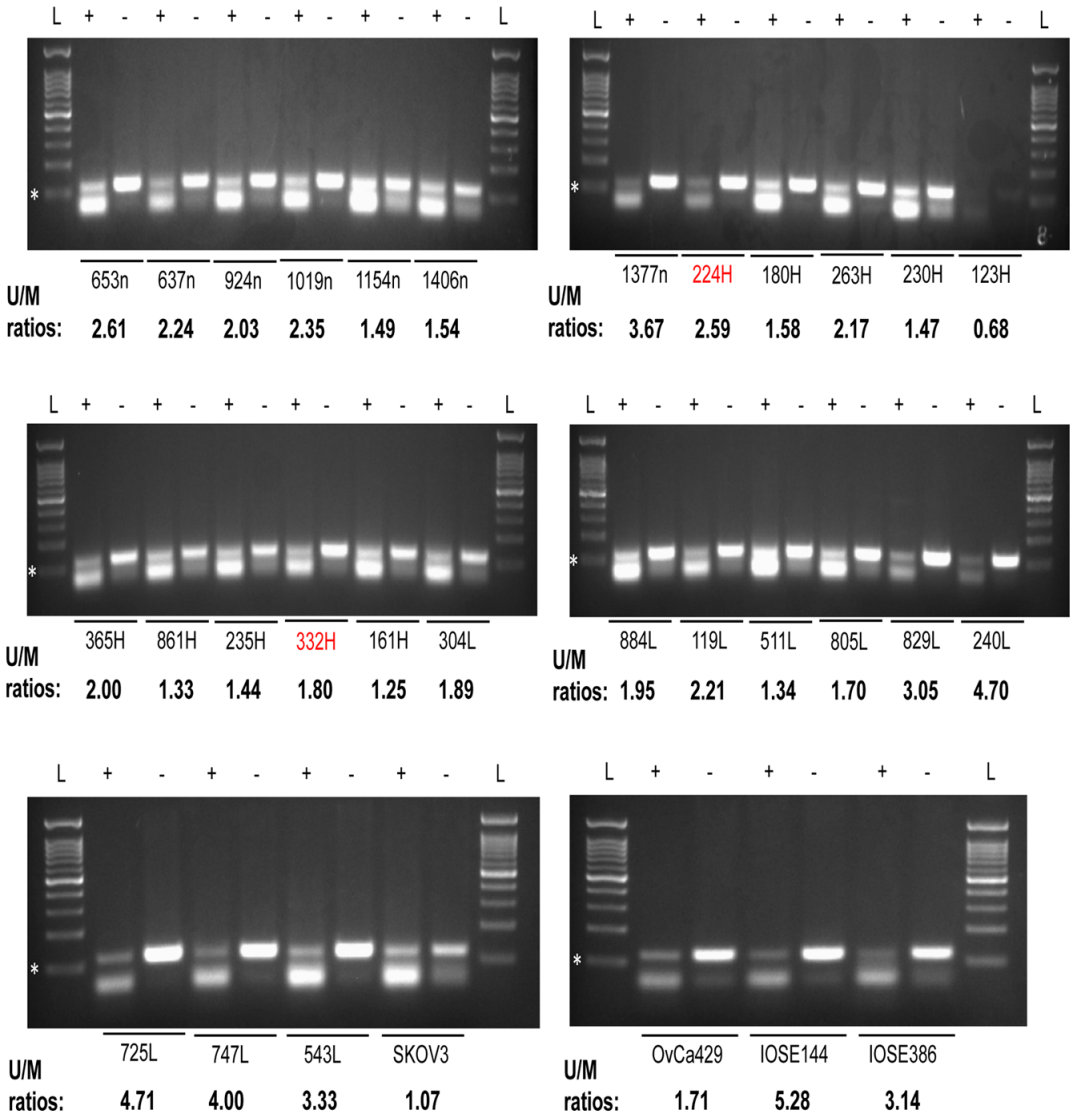
The KIF14 promoter is hypomethylated in primary OvCas, OvCa cell lines and normal tissues. Methylation-specific PCR of primary OvCa tumors and cell lines. L, molecular weight ladder; +, methylated primers; -, unmethylated primers; n, normal ovary tissue; H, *KIF14^HIGH^* tumor; L, *KIF14^LOW^* tumor; red, tumors with *KIF14* gain; U/M ratio, ratio of unmethylated product to methylated product (bold); white star, 100 bp molecular weight maker.

Epigenetic mechanisms of gene regulation, such as aberrant DNA methylation have been identified to upregulate oncogene expression and silence tumor suppressors in many cancers [18]. In OvCa tumors, DNA methylation patterns have been investigated as potential biomarkers for tumor progression and response to therapy [19]–[25]. To study *KIF14* promoter methylation, we documented the *KIF14* promoter region using the online software CpG Island Searcher (http://www.cpgislands.com). We identified a CpG island within the 4500 bp *KIF14* promoter region (−2371 to −1129) (Figure S1A), and designed methylation-specific primers (∼120 bp) against the largest CpG island using the online software Methprimer (Figure S1B). Interestingly the location of this major CpG island coincides with the above-analyzed *Sp1* and *YY1* binding sites (Figure S1A). In support of our findings, it is well known that Sp1 recognition sites are commonly located within CpG islands [26]. Bisulphite conversion of tumor DNA from 20 serous OvCa tumors (10 *KIF14* ^HIGH^ and 10 *KIF14* ^LOW^), 7 normal ovary tissues, and 4 OvCa cell lines (including 2 IOSE samples) was performed, followed by PCR using primers directed against methylated/unmethylated residues. Complete bisulphite conversion of the DNA was verified with the use of unmodified calponin-specific primers (Figure S2). Most tissue and cell line samples tested, including normal ovary controls, demonstrated higher abundance of unmethylated product than methylated product (Figure 6), as evidenced by the unmethylated/methylated (U/M) ratios above 1 (Table S4). Interestingly, analysis of the U/M ratios between *KIF14* ^HIGH^ and *KIF14* ^LOW^ samples revealed a statistically significant difference, wherein *KIF14* ^HIGH^ tumors demonstrated on average less unmethylated product (lower U/M means more methylated product) than *KIF14* ^LOW^ (higher U/M ratio means less methylated product; *P* = 0.009; Table S4). The methylation patterns in normal ovary were much more variable, and not statistically different from *KIF14* ^LOW^ tumors (*P* = 0.27) but approaching significance for *KIF14* ^HIGH^ tumors (*P* = 0.054). A marked difference was also seen in U/M ratios between OvCa cell lines and IOSE cells, following the same trend as for the *KIF14* ^HIGH^ vs *KIF14* ^LOW^ tumors; however sample size (n = 2) precluded statistical analysis. These results indicate that differential methylation patterns exist between OvCa tumors. Although in need of further investigation, the fact that unmethylated product is present in all normal and tumor tissues suggests a minor role for promoter methylation (of the particular region studied) in the control of *KIF14* overexpression in OvCa tumor cells.

### miR93, miR144 and miR382 expression are associated with KIF14 expression in primary OvCa tumors

**Figure 7:**
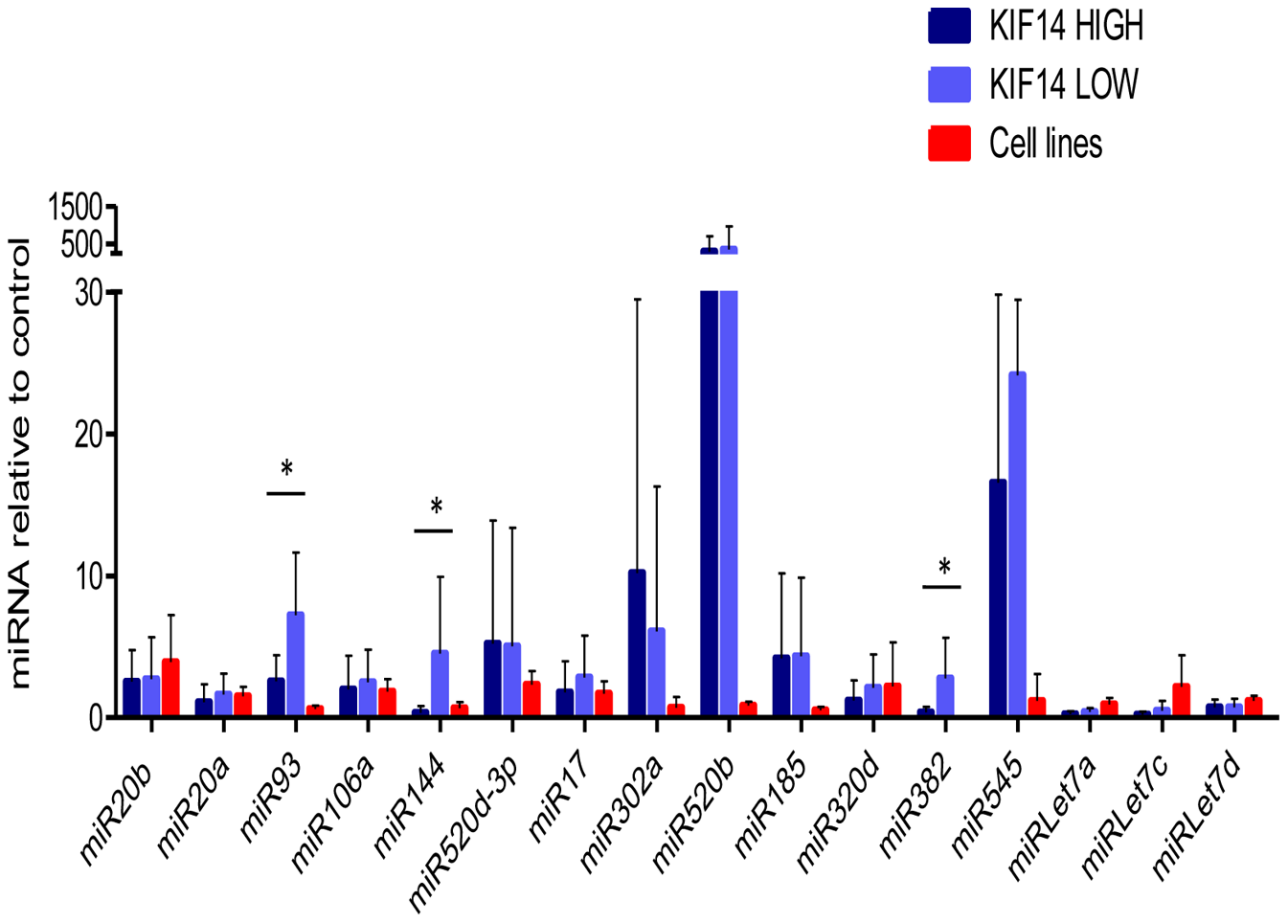
Candidate miRNA expression in primary OvCa tumors and OvCa cell lines. Mean miRNA expression in *KIF14* ^HIGH^ (dark blue) and *KIF14* ^LOW^ (light blue) primary serous OvCas, and established OvCa cell lines (red). Expression normalized to the endogenous control gene *RNU44*, relative to normal ovary tissues (primary OvCas) or IOSE (OvCa cell lines). * Significance, *P*<0.05, unpaired t-test. *P* = 0.002 (miR-93), *P* = 0.01 (miR-144), *P* = 0.006).

MicroRNAs (miRNAs) are a large, extensively studied group of small non-coding RNAs that downregulate protein expression by targeting mRNA translation and/or stability [27]. miRNAs have been implicated in the control of expression of many crucial cellular gene expression pathways in normal and cancer cells, and have demonstrated roles in ovarian cancer pathogenesis and progression [28]. To determine whether *KIF14* expression may be under miRNA control, we analyzed the *KIF14* gene region using 7 online miRNA binding site prediction tools (Table S5) that revealed multiple potential candidate miRNAs binding to the *KIF14* gene. The expression of 16 candidate miRNAs presenting one or more hits was studied on 6 OvCa cell lines (including 2 immortalized ovarian surface epithelium (OSE) controls) and 26 primary serous OvCa tumors; half (13) with low *KIF14* overexpression (*KIF14* ^LOW^), and half with high *KIF14* overexpression (*KIF14* ^HIGH^) [8]. Table S6 depicts miRNA expression of the 16 selected miRNAs for *KIF14* ^HIGH^ and *KIF14* ^LOW^ (depicted by sample number, followed by H for HIGH, L for LOW) serous OvCa tumors, and OvCa cell lines. miRNA expression was normalized to the endogenous control gene *RNU44*, and fold change was calculated in relation to mean expression of normal tissues (a mix of normal ovary and tubal epithelial tissues) for primary OvCa tumors, and mean expression of IOSE cells for OvCa cell lines. Three of the 16 selected miRNAs emerged as potential regulatory candidates, displaying significantly lower expression in *KIF14* ^HIGH^ OvCa tumors in relation to *KIF14* ^LOW^ OvCa tumors (highlighted in yellow): miR93, miR144, and miR382. Two-tailed, unpaired t-test analysis revealed that all three candidates displayed significantly lower miRNA expression in *KIF14* ^HIGH^ vs. *KIF14* ^LOW^ OvCa tumors (Table S6, *P*<0.01). Mean miRNA expression in *KIF14* ^HIGH^ and *KIF14* ^LOW^ tumors and OvCa cell lines with standard deviation is presented in Figure 7. Pearson correlation analysis comparing miRNA expression levels of miR93, miR144 and miR382 with *KIF14* mRNA expression in these same tumors revealed a significant negative correlation between miRNA and *KIF14* expression levels, indicating that high *KIF14* overexpression in OvCa tumors might be a consequence of the relatively low abundance of these candidate miRNAs (Table S6, *P*<0.04). The expression profile of the candidate miRNAs was also confirmed in OvCa cell lines in comparison to IOSE cells (Figure 7 and Table S6).

### miR93, miR144 and miR382 modulate *KIF14* mRNA expression in an OvCa cell line

**Figure 8:**
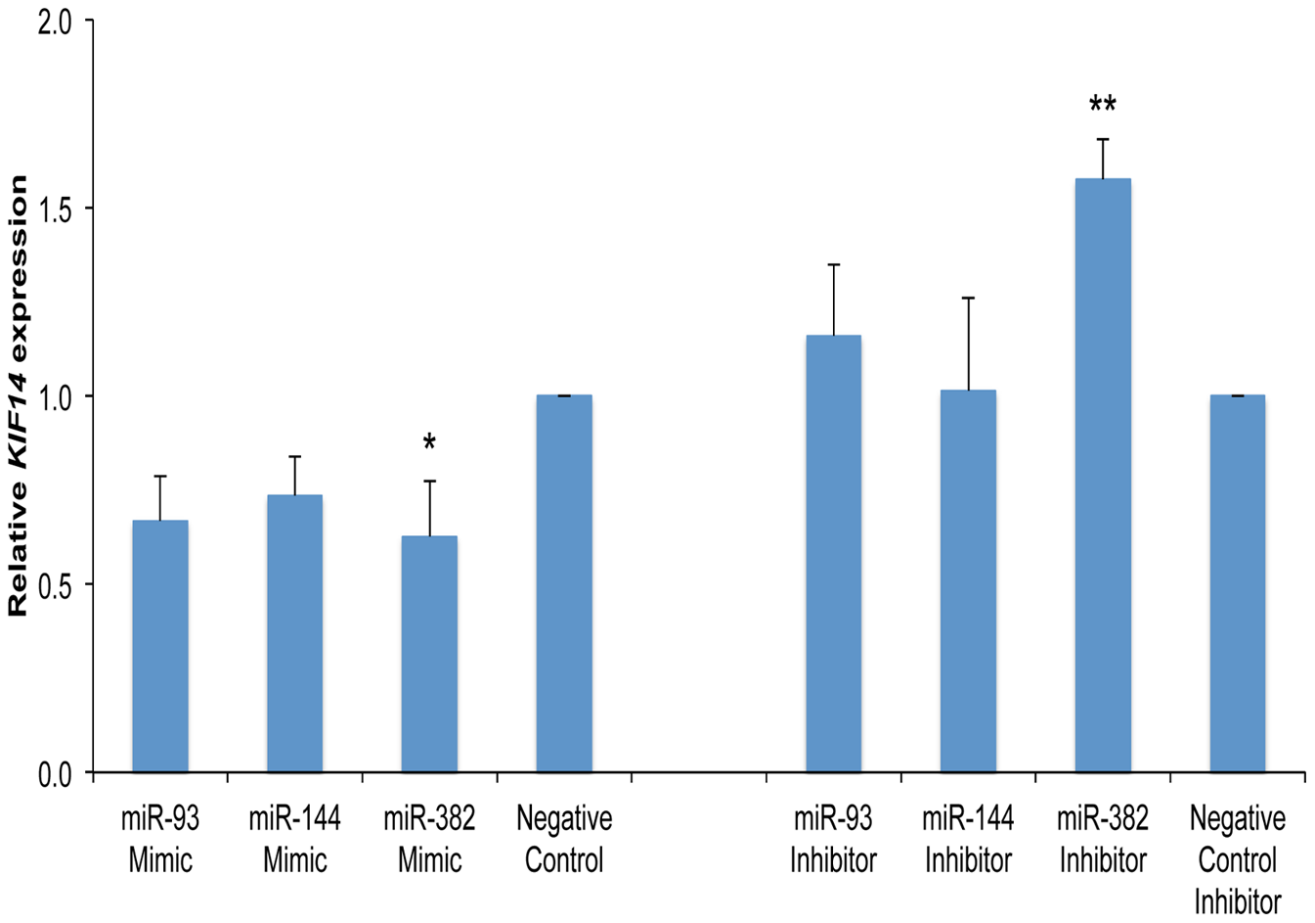
Candidate miRNAs modulate *KIF14* mRNA expression in an OvCa cell line. SKOV3 cells were treated with either candidate miRNA mimics that increased miRNA expression 200–6000-fold, or miRNA inhibitors that decreased expression by 70–100%. *KIF14* mRNA expression was measured using real time PCR. Graph represents *KIF14* expression relative to negative control transfections. Average ± SD of three separate experiments shown. Significance: *, *P*<0.05; **, *P*<0.01 compared with control, one-way ANOVA with Dunnett's post hoc tests.

To determine whether the identified candidate miRNAs could modulate *KIF14* mRNA expression, we treated SKOV3 cells with either the candidate miRNA mimic or inhibitor, and measured *KIF14* mRNA expression via real time PCR. miR93 and miR144 mimics caused a modest but reproducible decrease in *KIF14* mRNA expression that did not achieve statistical significance, while treatment with miR382 mimic resulted in a significant inhibition of *KIF14* mRNA expression (Figure 8). Correspondingly, treatment with miR93 inhibitor resulted in a modest increase in *KIF14* mRNA expression (Figure 8), while treatment with a miR382 inhibitor produced a substantial increase in *KIF14* mRNA expression (Figure 8). These results point to the potential that these candidate miRNAs (especially miR382) are modulators of *KIF14* mRNA levels in OvCas that could contribute to the overexpression of *KIF14* mRNA in OvCa tumors.

## Discussion

We and others identified *KIF14* as an important oncogene, prognostic indicator, biomarker and therapeutic target in the progression of multiple cancers, including retinoblastoma, glioma, breast, lung, renal, hepatocellular and ovarian cancers [1], [4]–[9],[14],[29]–[32]]. *KIF14* mRNA is highly overexpressed in the majority of OvCa tumors [8], and has recently been identified as a marker for taxane resistance in a small cohort of OvCa tumors [33]. Thus insight into the regulation of its overexpression is crucial for developing therapeutic approaches to reduce *KIF14* expression in OvCa cells. We have previously shown that a proportion of OvCa tumors demonstrate genomic gain of *KIF14* (up to 30%, depending on histological subtype) that correlates with high mRNA overexpression, indicating genomic gain as one mechanism of KIF14 overexpression. Since over 90% of OvCas overexpress *KIF14* mRNA, other mechanisms of overexpression are likely to exist within these tumors. The goal of this study was to characterize additional mechanisms of KIF14 overexpression in OvCa cells. This type of comprehensive gene regulation analysis is important prior to development of new therapeutic strategies.

We first characterized the regulation of the *KIF14* promoter region in OvCa cell lines. We identified via luciferase reporter activity a *cis*-regulatory region within the ∼4500 bp promoter region to harbour putative binding sites for the transcription factors *Sp1*, *HSF1*, and *YY1*. Through transient siRNA knockdown of these transcription factors in OvCa cell lines, we determined that inhibition of *Sp1* and *YY1*, but not *HSF1*, significantly decreased *KIF14* mRNA expression. Furthermore, we show that both endogenous Sp1 and YY1 can bind to the *KIF14* promoter in OvCa cell lines, indicating their potential importance in OvCa progression. The consensus binding site for the Sp1 family of transcription factors is GGGGCGGGG. In our analysis of the *KIF14* promoter region, only two partial Sp1 binding sites were found within the putative *cis* regulatory region; the siRNA, ChIP and expression data confirm a regulatory role for Sp1 in enhancing *KIF14* expression, similar to previous data showing Sp1 regulation of the *MCAK* promoter [15]. Sp1 is a ubiquitously expressed transcription factor that is involved in modulating normal and tumorigenic gene expression in multiple cancers. It is overexpressed in breast, thyroid, hepatocellular, prostate, pancreatic, gastric, lung and ovarian cancers [34**]**–[37], and small molecule inhibitors of Sp1 have been tested in pre-clinical models with successful reduction in tumor burden [38], [39]. Our data demonstrate not only that Sp1 can enhance *KIF14* expression in OvCa cell lines, but that Sp1 is overexpressed and correlates with high *KIF14* overexpression in primary OvCa tumors. Targeting Sp1 expression may reduce KIF14 levels in tumor cells, and could contribute to a therapeutic strategy against OvCa progression.

YY1 is a transcription factor that also has documented roles in either enhancing or repressing gene expression in multiple cancers [40**]**–[42], however its tumorigenic overexpression and prognostic value have been the subject of debate due to conflicting reports for multiple cancers, including in breast, colon, prostate, osteosarcoma, cervical and ovarian cancers [42]. Interestingly, YY1 has been shown to interact with general transcription factors such as Sp1 [43**]**–[47] to enhance gene expression. YY1 can bind to DNA and directly modulate transcriptional activity, and also act as a cofactor to recruit into an active transcriptional complex other factors, including Sp1 [42]. Based on our data, one can easily envision YY1 and Sp1 cooperating to enhance KIF14 overexpression in OvCa tumors. Detailed mechanisms of their interaction remain to be investigated.

HSF1 is a heat-shock response transcription factor that plays a tumorigenic role in multiple cancers [48]. Although we found HSF1 binding sites within the identified *cis* regulatory region of the *KIF14* promoter, our data demonstrate no effect of *HSF1* knockdown on KIF14 gene or protein expression, and very weak endogenous binding of HSF1 to the *KIF14* promoter in OvCa cell lines.

Methylation analysis of the largest CpG island within the human *KIF14* promoter revealed that the *KIF14* promoter is largely hypomethylated in OvCa primary tumors, normal ovary tissues, and cell lines, indicating that methylation may not be a mechanism regulating *KIF14* overexpression within OvCa tumors. This result is not surprising, as the *KIF14* gene is crucial in normal and tumor cells to control the last stages of cytokinesis [12], [13], and thus there is most likely a need for some expression (albeit low in normal tissues) to maintain normal proliferation and viability. However, the presence of more methylated product in *KIF14^HIGH^* tumors than *KIF14^LOW^* tumors indicates that a differential methylation pattern may exist between low and high KIF14 tumors. Although warranting further investigation given that promoter methylation is generally seen as a mechanism of transcriptional repression [18], these results raise the intriguing possibility of a feedback mechanism, attempting, but inadequate to reduce KIF14 levels in tumors with high KIF14 expression. Although the other minor CpG islands were not tested, our results indicate that other mechanisms of gene regulation, such as genomic gain, transcriptional activation, or miRNA regulation are more likely to control *KIF14* overexpression in serous OvCa tumors.

The knockdown of *YY1* produced a much larger decrease in KIF14 protein than mRNA, which prompted us to investigate the possibility that KIF14 could be regulated by miRNAs. Through miRNA binding site prediction software, we identified 16 putative miRNAs to bind to the *KIF14* promoter region. Expression analysis of these miRNAs in OvCa cell lines and primary OvCa tumors in comparison to normal ovary tissues and IOSE cells revealed that miR93, miR144 and miR382 were expressed significantly lower in *KIF14* ^HIGH^ primary OvCa tumors than in *KIF14* ^LOW^ tumors. These miRNAs were also expressed lower in established OvCa cell lines compared to IOSE cells, suggesting that these miRNAs are interesting potential regulators of *KIF14* levels in the promotion of OvCa tumor progression worthy of further characterization. We determined that treatment of SKOV3 cells with candidate miRNA inhibitors specifically increased *KIF14* mRNA expression. Although the role of these candidate miRNAs in the modulation of *KIF14* protein levels has yet to be assessed, our results clearly implicate these miRNAs in the maintenance of *KIF14* mRNA levels, and potential KIF14 overexpression in OvCa tumors. Interestingly, the largest increase in *KIF14* mRNA expression resulted from miR382 inhibitor treatment; this candidate miRNA also showed the strongest inverse correlation with *KIF14* mRNA expression in primary OvCa tumors, the lowest level of *KIF14* mRNA expression in OvCa cell lines, and the greatest decrease in *KIF14* mRNA expression when overexpressed. Our results thus highlight the potential importance of miR382 in the progression of OvCa tumors.

miR93 has been recently shown to have a role modulating cisplatin resistance in OvCa cell lines by targeting the PTEN tumor suppressor [49]. This study was conducted in established OvCa cell lines treated with chemotherapy, as opposed to the chemo-naïve primary OvCa tumors for miRNA expression analysis in our study. Whether miR93 plays a role in modulating cisplatin resistance in primary OvCa tumors remains to be determined, however our results suggest that loss of miR93 may modulate *KIF14* mRNA overexpression in primary OvCa tumor cells to promote poor outcome. To date, miR144 has no documented role in OvCa tumors, but low miR144 levels are independently prognostic of poor outcome in primary colorectal cancers [50]. Decrease in miR382 expression has been correlated with poor outcomes in osteosarcomas [51]. miR382 is located at the 14q32 locus, which has been shown to be lost in primary OvCa tumors [52]; our results are the first evidence confirming miR382 as a novel oncogenic regulator within that region.

Only serous OvCa tumors were selected for methylation and miRNA analysis because i) serous OvCa tumors represent the most common histological subtype, and ii) *KIF14* mRNA expression was predictive of poor outcome only in the serous subtype [8]. The number of samples (10 for methylation, 13 for miRNA analyses within each expression group) was studied because i) ample genetic material (both DNA and RNA) was available, and ii) these tumors had complete clinical outcome datasets (including progression-free and overall survival data). As controls, two samples with *KIF14* genomic gain (224H and 332H) were included in both the methylation and miRNA analyses. 332H showed very low miRNA expression despite genomic gain, suggesting that multiple mechanisms (genomic gain and high *KIF14* transcriptional activity) may be at play within the same tumor to ensure *KIF14* overexpression.

We have identified genomic, transcriptional and miRNA regulation as potential mechanisms of *KIF14* overexpression in OvCa cell lines and primary tumor tissues. To our knowledge, this is the first report documenting the regulation of *KIF14* overexpression in primary tumors. However, the functional implication of these regulators in tumorigenesis needs more investigation. The limited number of samples employed in our studies did not allow for any predictive analysis of transcription factor or candidate miRNA expression with respect to patient outcome. Nonetheless, our data identify multiple mechanisms of *KIF14* regulation in OvCa tumors, and provide insight into potentially novel avenues of therapeutic intervention to regulate *KIF14* expression in tumor cells. These results do not preclude the possibility that the overexpression of *KIF14* in OvCa tumors may be regulated via additional mechanisms (i.e., histone modifications and expression of histone modifying enzymes) [53], or that multiple mechanisms may be at play to drive *KIF14* overexpression within the same tumor.

The dismal prognosis associated with many solid tumors arises from poorly effective therapies for treatment of recurrent disease. The identification of potential regulatory transcription factors and miRNAs will open the door for development of more precise, less invasive methods to predict outcome, as miRNAs especially are known to be stably detected in serum [54]; these candidate tumor miRNAs could be developed into non-invasive detection and/or prognostic markers. With further validation, these putative transcriptional regulators may be targets for selective anti-KIF14 therapy.

## Supporting Information

Figure S1 **Methylation specific PCR.** **A** CpG island analysis of the KIF14 promoter identified one CpG island between −2371 to −1129 (1243 bp). Blue line delineates the CpG island, while red lines represent potential methylated CpG residues. **B** Design of methylation-specific primers for the KIF14 promoter.
(TIF)

Figure S2 **Calponin PCR.** Calponin PCR of primary OvCa tumors and cell lines. L, molecular weight ladder; n, normal ovary tissue; H, *KIF14^HIGH^* tumor; L, *KIF14^LOW^* tumor; red, tumors with *KIF14* gain.
(TIF)

Figure S3 **SP1 and YY1 bind endogenously to the human KIF14 promoter in OvCa cell lines.** ChIP assays of endogenous YY1, Sp1 and HSF1 with the *KIF14* promoter region (−2150 to −2366 = 216 bp amplicon, black arrow) in cell lines SKOV3, OvCa429, and WERI-Rb1 (positive control for Sp1 binding), compared to IgG (negative control and RNA pol II (positive control for ChIP, 250 bp amplicon). Numbers represent relative expression values (normalized to RNA pol II, relative to IgG) Black asterisk, 200 bp marker.
(TIF)

Figure S4 **KIF14 mRNA expression correlates with SP1 and YY1 expression in OvCa tumors without genomic gain.** Pearson correlation analysis between KIF14 gain (**A–C**) or no gain (**D–F**) OvCa tumors and SP1 (**A, D**), HSF1 (**B, E**) and YY1 (**C, F**) mRNA expression. r =  correlation coefficient; *P* significance at <0.05; N =  number of samples.
(TIF)

Table S1 **Primer sequences for PCR of KIF14 promoter fragment.**
(TIF)

Table S2 **Target sequences for siRNAs.**
(TIF)

Table S3 **Sequences for miRNA specific assays.**
(TIF)

Table S4 **Unmethylated/Methylated DNA ratios.**
(TIF)

Table S5 **miRNA database predictions for miRNA candidates binding to the KIF14 promoter.**
(TIF)

Table S6 **miRNA expression analyses of primary OvCas and OvCa cell lines.** Highlighted miRNAs represent significant difference in expression between *KIF14* ^HIGH^ (H) and *KIF14* ^LOW^ (L) OvCas. AVG, average, STDEV, standard deviation. t-test, comparison of miRNA expression between *KIF14* ^HIGH^ and *KIF14* ^LOW^ groups. Pearson correlation, comparison of miRNA expression to *KIF14* expression in the entire tumor cohort. *P*<0.05, significant difference.
(TIF)
