# Supplementary figures and images for: Transcriptional and Epigenetic Regulation of KIF14 Overexpression in Ovarian Cancer
Source: PLoS One. 2014 Mar 13;9(3):e91540. doi: 10.1371/journal.pone.0091540 (PMC3953446; doi:10.1371/journal.pone.0091540)

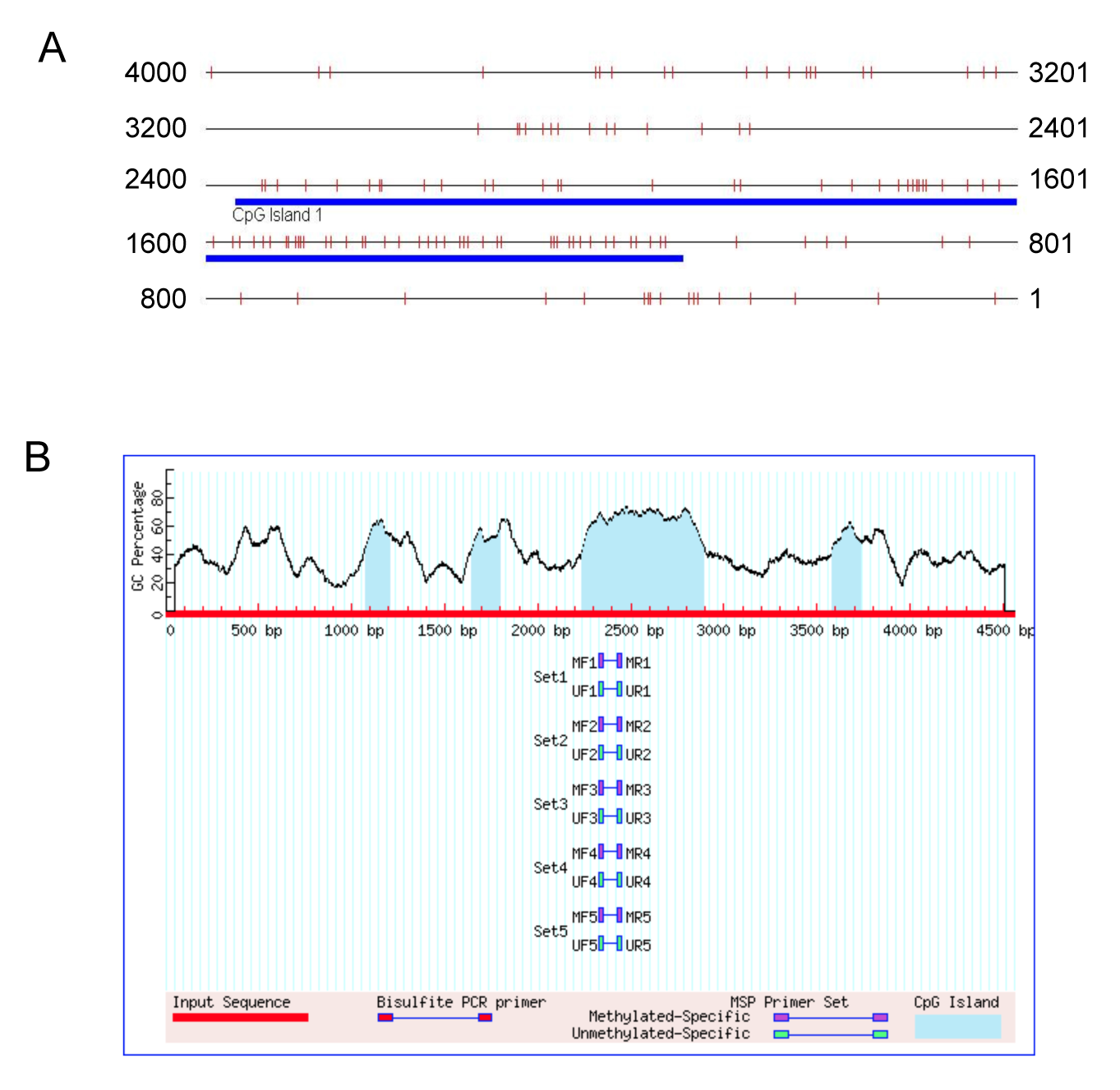

Supplement: Figure S1 — Methylation specific PCR. A CpG island analysis of the KIF14 promoter identified one CpG island between −2371 to −1129 (1243 bp). Blue line delineates the CpG island, while red lines represent potential methylated CpG residues. B Design of methylation-specific primers for the KIF14 promoter. (TIF) [file pone.0091540.s001.tif]

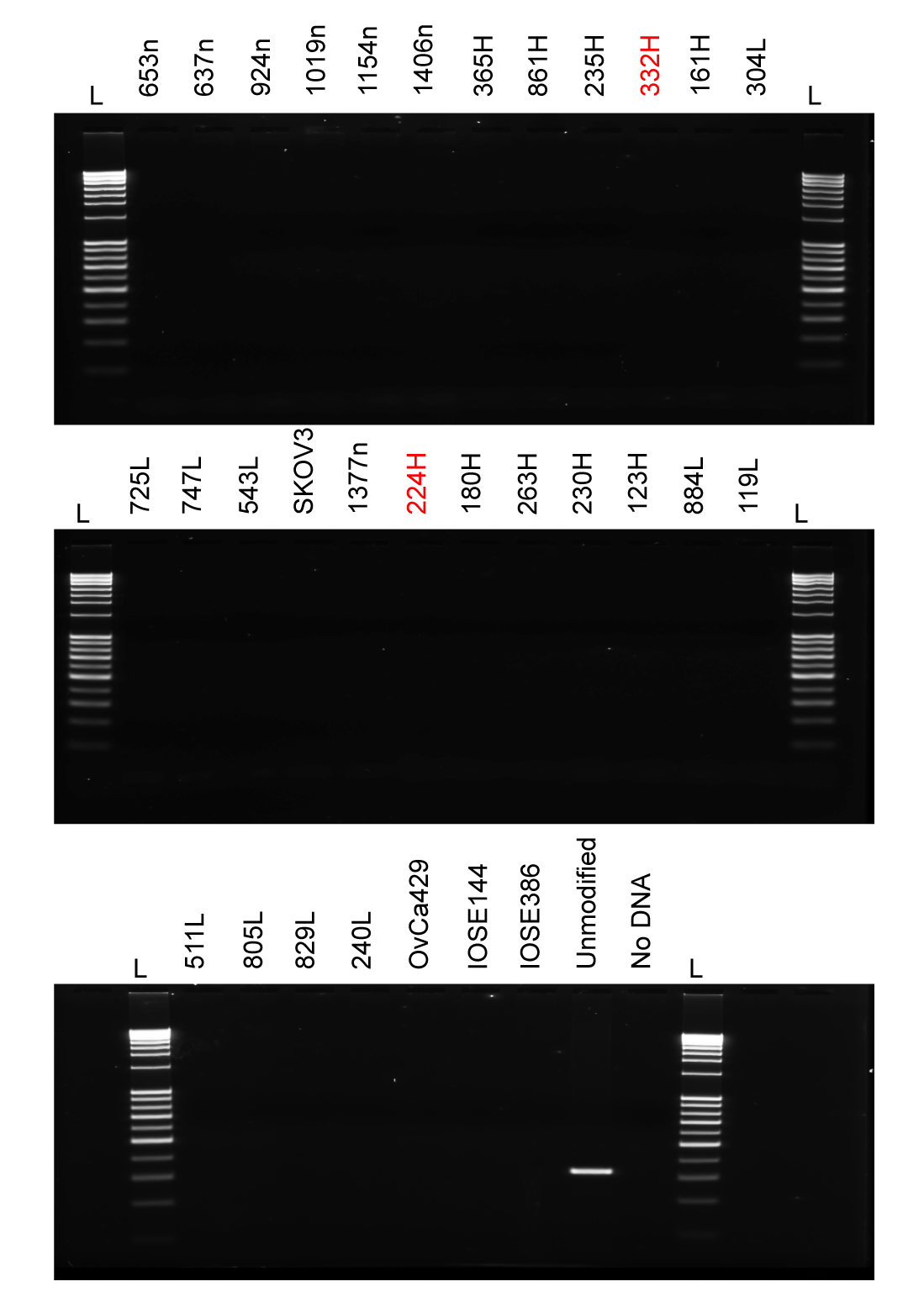

Supplement: Figure S2 — Calponin PCR. Calponin PCR of primary OvCa tumors and cell lines. L, molecular weight ladder; n, normal ovary tissue; H, KIF14HIGH tumor; L, KIF14LOW tumor; red, tumors with KIF14 gain. (TIF) [file pone.0091540.s002.tif]

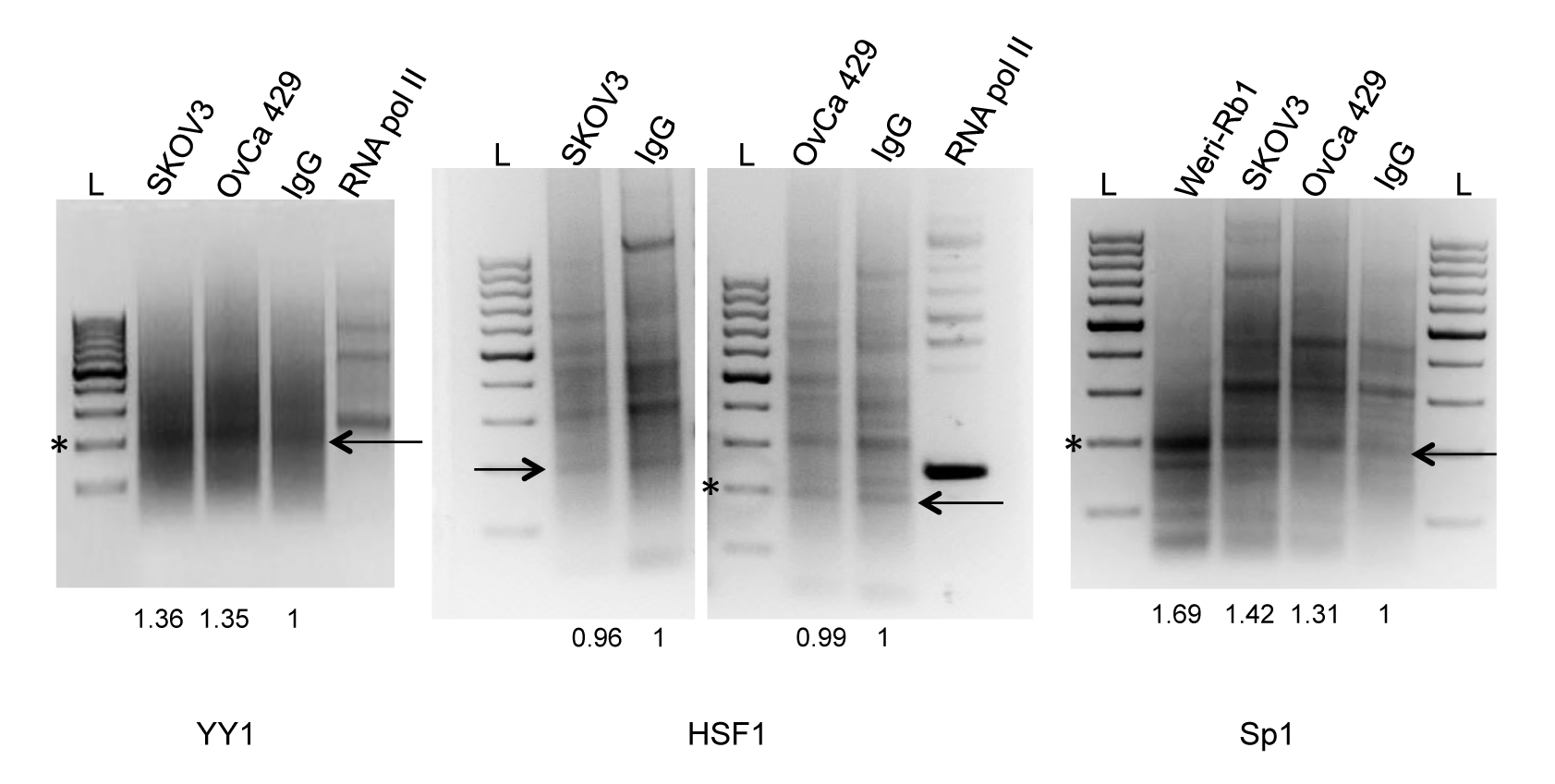

Supplement: Figure S3 — SP1 and YY1 bind endogenously to the human KIF14 promoter in OvCa cell lines. ChIP assays of endogenous YY1, Sp1 and HSF1 with the KIF14 promoter region (−2150 to −2366 = 216 bp amplicon, black arrow) in cell lines SKOV3, OvCa429, and WERI-Rb1 (positive control for Sp1 binding), compared to IgG (negative control and RNA pol II (positive control for ChIP, 250 bp amplicon). Numbers represent relative expression values (normalized to RNA pol II, relative to IgG) Black asterisk, 200 bp marker. (TIF) [file pone.0091540.s003.tif]

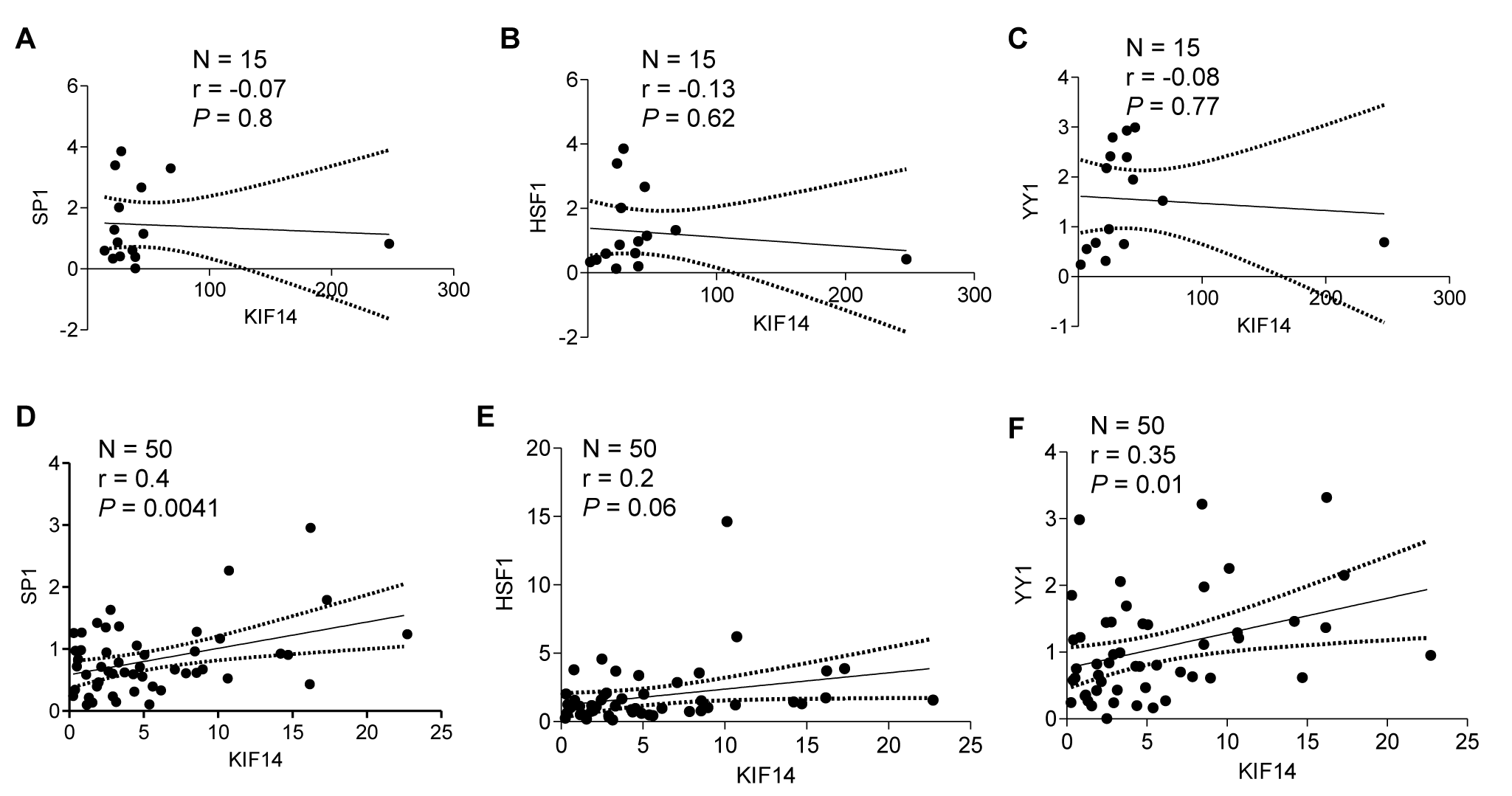

Supplement: Figure S4 — KIF14 mRNA expression correlates with SP1 and YY1 expression in OvCa tumors without genomic gain. Pearson correlation analysis between KIF14 gain (A–C) or no gain (D–F) OvCa tumors and SP1 (A, D), HSF1 (B, E) and YY1 (C, F) mRNA expression. r = correlation coefficient; P significance at <0.05; N = number of samples. (TIF) [file pone.0091540.s004.tif]

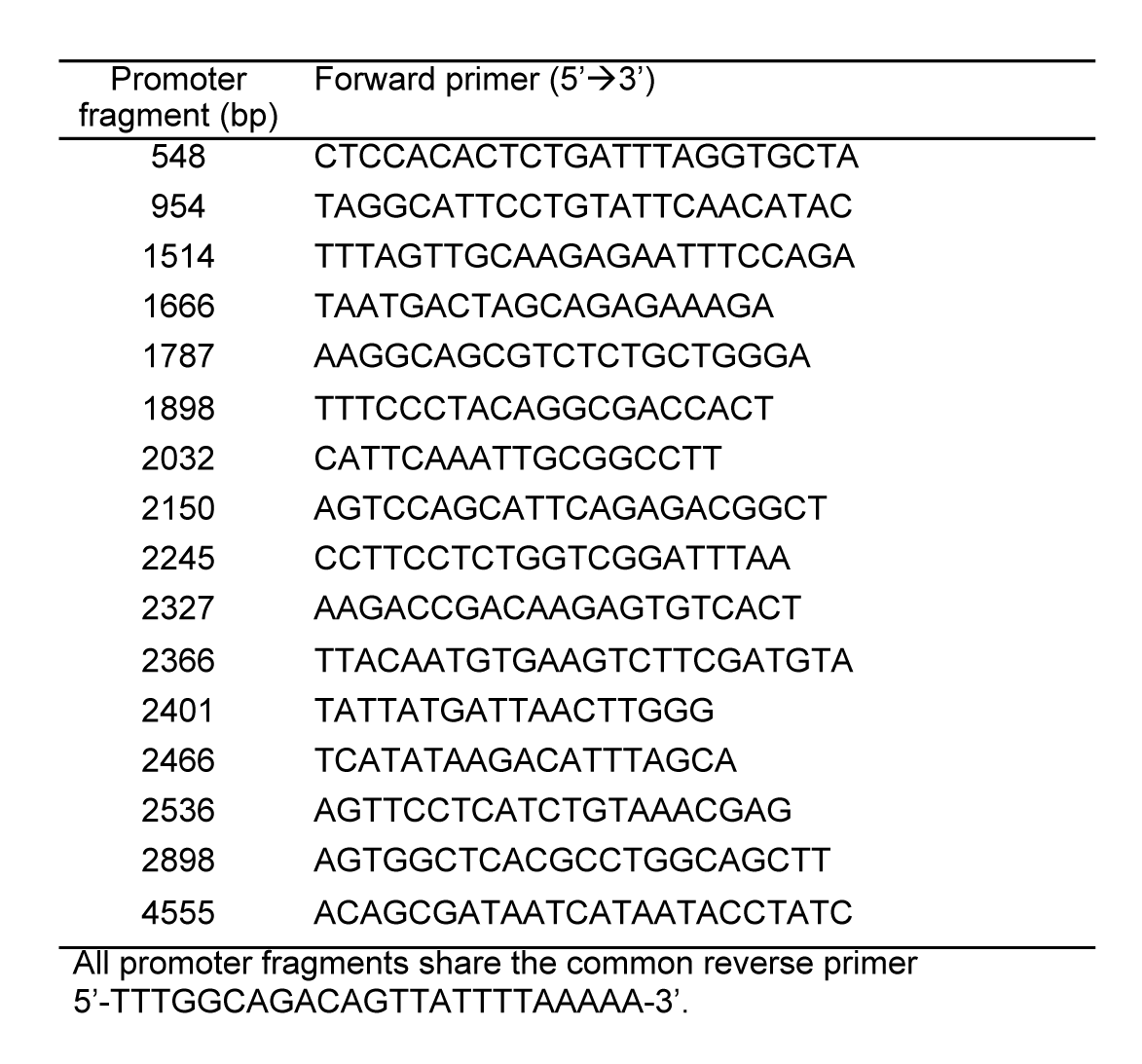

Supplement: Table S1 — Primer sequences for PCR of KIF14 promoter fragment. (TIF) [file pone.0091540.s005.tif]

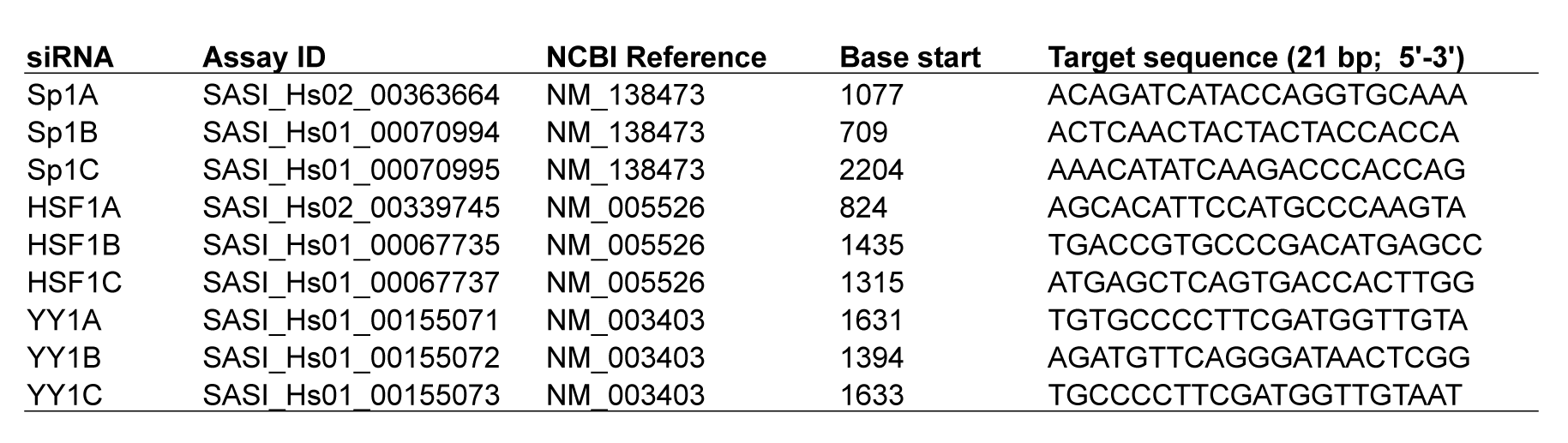

Supplement: Table S2 — Target sequences for siRNAs. (TIF) [file pone.0091540.s006.tif]

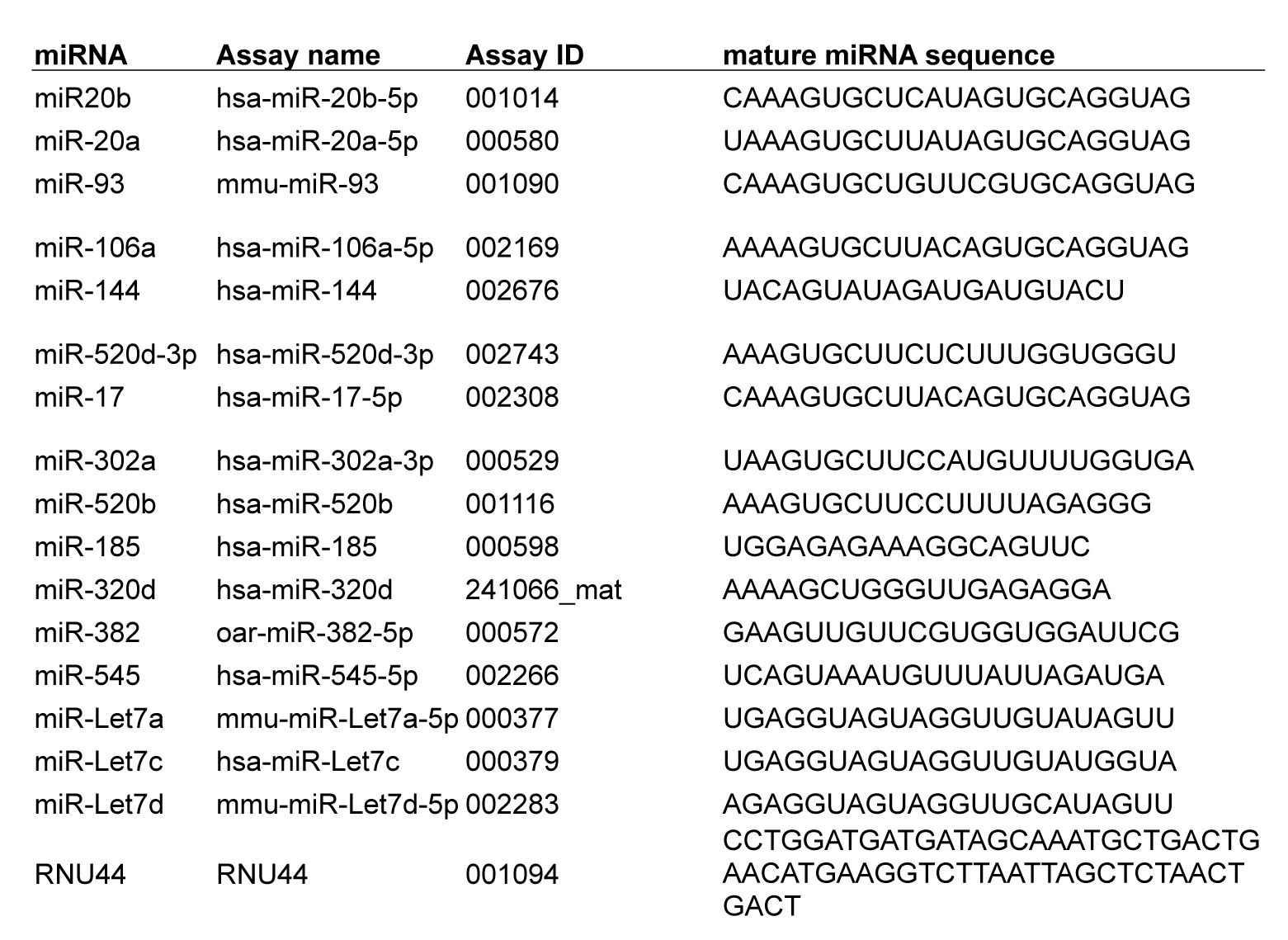

Supplement: Table S3 — Sequences for miRNA specific assays. (TIF) [file pone.0091540.s007.tif]

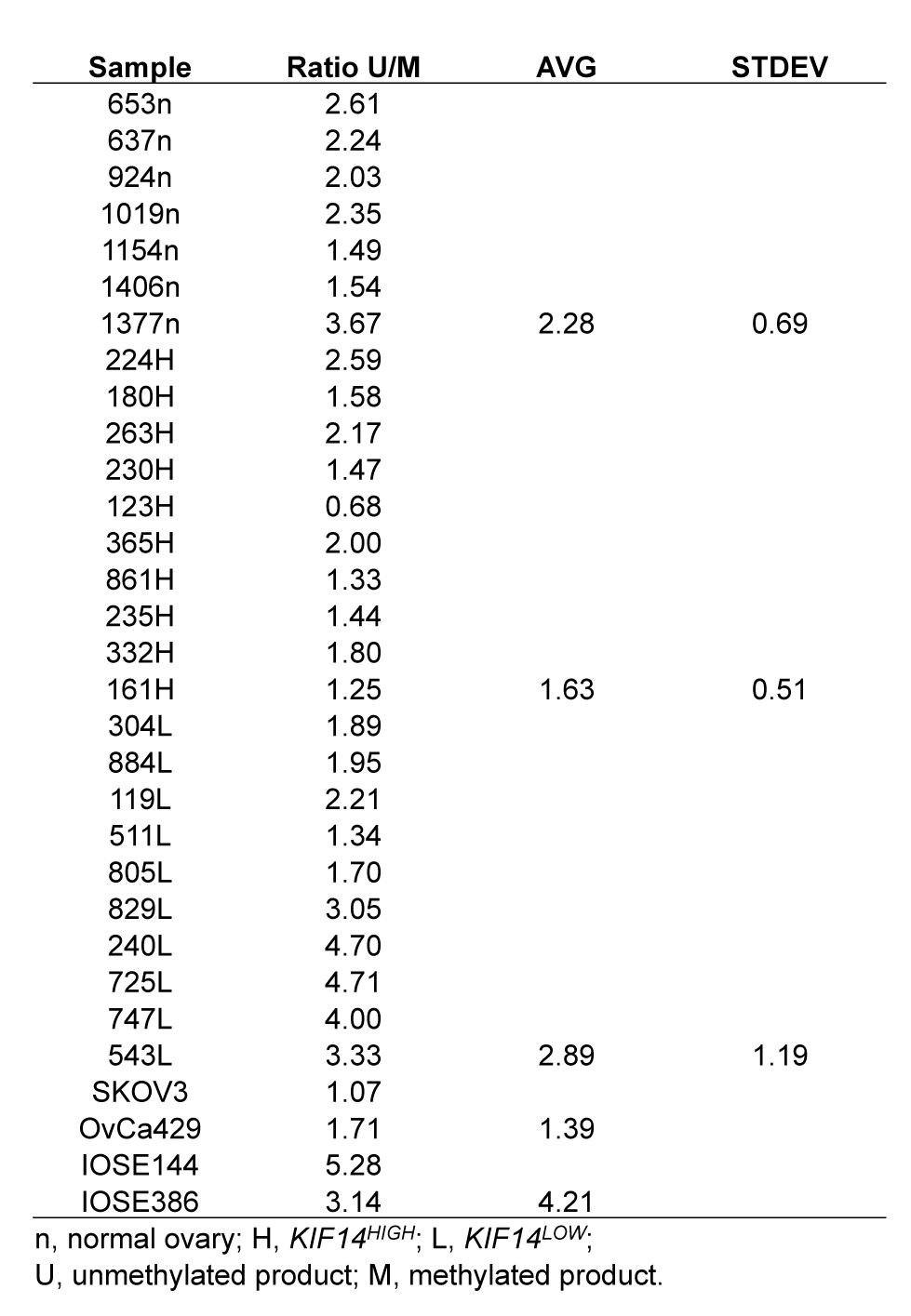

Supplement: Table S4 — Unmethylated/Methylated DNA ratios. (TIF) [file pone.0091540.s008.tif]

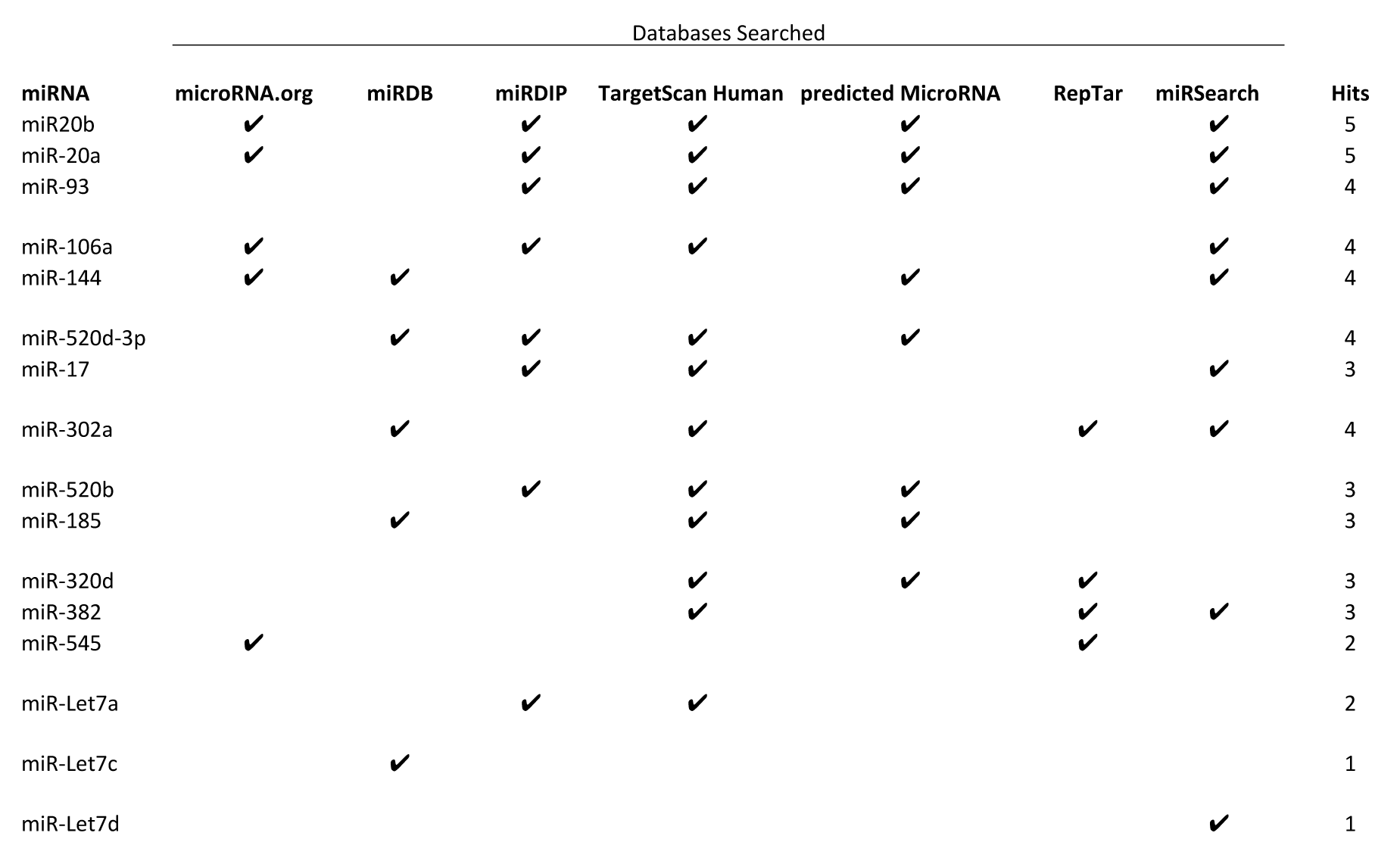

Supplement: Table S5 — miRNA database predictions for miRNA candidates binding to the KIF14 promoter. (TIF) [file pone.0091540.s009.tif]

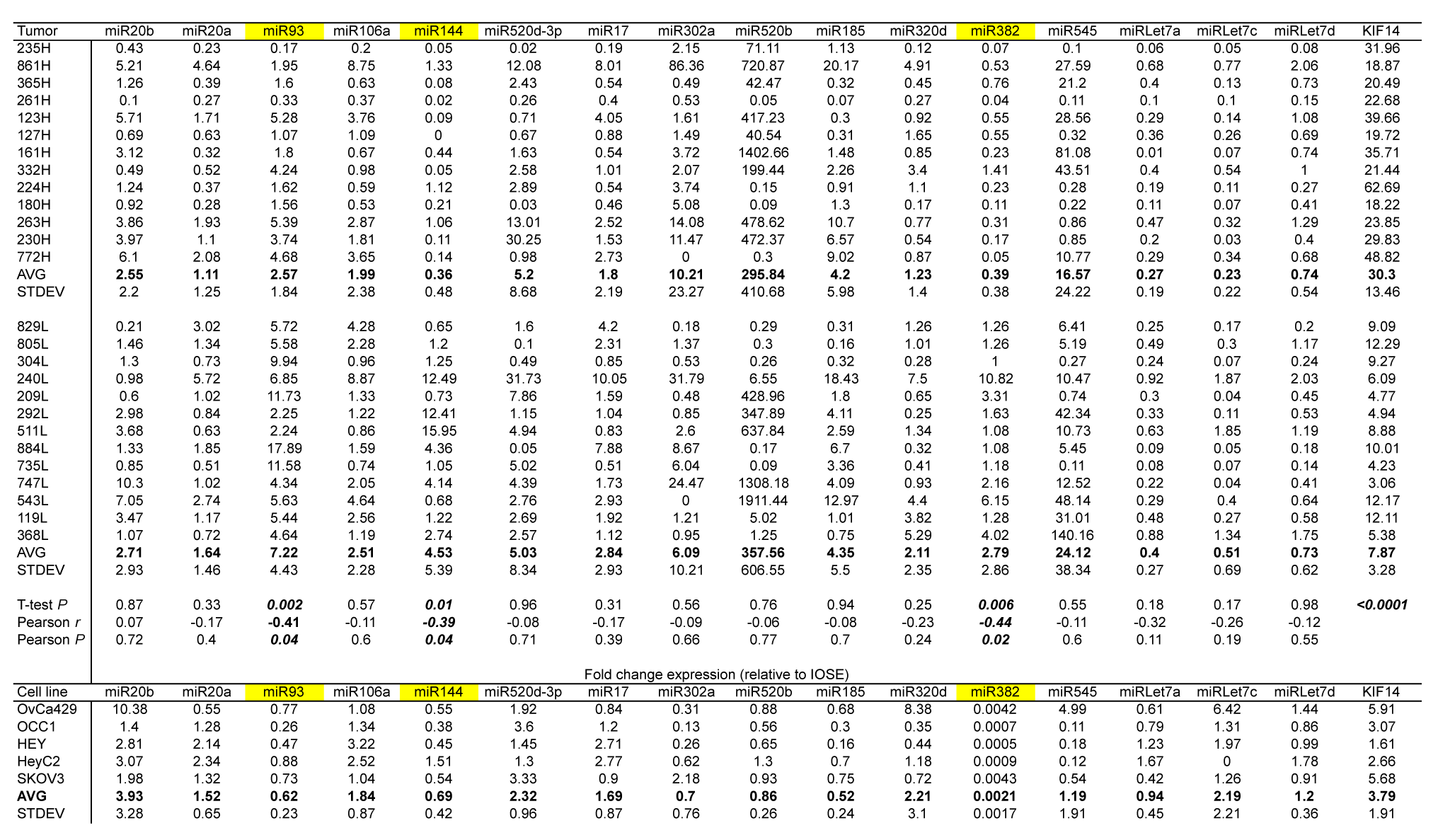

Supplement: Table S6 — miRNA expression analyses of primary OvCas and OvCa cell lines. Highlighted miRNAs represent significant difference in expression between KIF14 HIGH (H) and KIF14 LOW (L) OvCas. AVG, average, STDEV, standard deviation. t-test, comparison of miRNA expression between KIF14 HIGH and KIF14 LOW groups. Pearson correlation, comparison of miRNA expression to KIF14 expression in the entire tumor cohort. P<0.05, significant difference. (TIF) [file pone.0091540.s010.tif]
